# Parenting experiences and family dynamics in pediatric burn care settings from hospitalization to the return home: a scoping review

**DOI:** 10.3389/fpsyg.2026.1897592

**Published:** 2026-09-23

**Authors:** Elisabete Cioga, Cristina Costeira, Nuno Correia, Filipa Ventura, Dulce Cruz, Carlos Laranjeira

**Affiliations:** 1 Comprehensive Health Research Centre (CHRC), University of Évora, Évora, Portugal; 2 School of Health Sciences, University of Leiria and Oeste, Leiria, Portugal; 3 Centre for Innovative Care and Health Technology (ciTechCare), University of Leiria and Oeste, Leiria, Portugal; 4 Health Sciences Research Unit: Nursing, Nursing School of University of Coimbra, Coimbra, Portugal; 5 Nursing School of University of Coimbra, Coimbra, Portugal; 6 São João de Deus Higher School of Nursing, University of Évora, Évora, Portugal

**Keywords:** adjustment, burns, pediatric, family, hospitalization, parents

## Abstract

**Introduction:**

Pediatric burns constitute a traumatic event for the child and the family, particularly for parents who assume a continuous and central role in care. Despite a growing body of research on pediatric burns, knowledge about parental experiences remains fragmented. This scoping review aims to map the available evidence on the experience of parents and carers of children with burns across the continuum of care, focusing on traumatic impact, coping responses, and continuity-of-care challenges.

**Methods:**

This study followed the methodology proposed by the Joanna Briggs Institute (JBI) guidelines and structured using the PRISMA-ScR. Studies were identified through MEDLINE, CINAHL, Academic Search Complete, Psychology and Behavioral Sciences Collection, PsycINFO, Cochrane Library, Scopus, and Web of Science Core Collection. The inclusion criteria considered studies related to the psychological and emotional impact associated with pediatric burns and hospitalization on parental and family dynamics.

**Results:**

A total of 63 studies published between 1993 and 2025 were included. They describe parental experiences across the continuum of pediatric burn care as characterized by intense emotional and functional demands arising from the immediate emotional impact of the injury, prognostic uncertainty, fear, and early disruptions to family organization. Parental adjustment is described as a non-linear and fluctuating process, marked by continuous attempts to regain control, restore parental identity, and rebuild family functioning.

**Conclusion:**

This scoping review mapped the available evidence on parental adjustment, described as an emotionally intense and functionally demanding process, shaped by interactions among family members, the healthcare team, and community systems. Future priorities should include multicenter longitudinal studies to map adjustment trajectories and identify predictors of recovery and post-traumatic growth.

## 1 Introduction

Burn injuries in childhood remain among the most severe causes of pediatric morbidity, affecting physical integrity but also psychological wellbeing and family functioning. Globally, burns are responsible for an estimated 180,000 deaths annually, mostly in low- and middle-income countries, and remain a leading cause of non-fatal morbidity (Smolle et al., 2017). Children account for nearly 42% of all burn cases worldwide, with more than half occurring in children aged 1–5 years. Although many pediatric burns are managed in ambulatory settings, more severe burn injuries require prolonged hospitalization, surgical intervention, and rehabilitation (Jordan et al., 2022). Beyond physical injury, pediatric burns represent a complex traumatic event with far-reaching implications for parents and carers (Atapattu et al., 2024; Wickens et al., 2024).

During hospitalization, parents frequently experience intense emotional distress, manifested as shock, fear, helplessness, and guilt, exacerbated by uncertainty regarding prognosis and recovery (Egberts et al., 2020). Previous studies have reported elevated levels of anxiety, depressive symptoms, and post-traumatic stress symptoms (PTSS) among parents of children with burn injuries, with some studies also documenting clinically significant post-traumatic stress disorder (PTSD) symptoms that may persist beyond hospital discharge (Huo et al., 2024; Townsend et al., 2023; Wickens et al., 2024). Parents also face disruptions to family routines and professional responsibilities, which intensify feelings of isolation and burden (Huo et al., 2024; Lernevall et al., 2023; Santos et al., 2024). These challenges are even more pronounced in low-resource settings, where socioeconomic constraints further compromise parental adjustment (Khan et al., 2024). Although spirituality, social support, and active involvement in care may foster resilience, many families report unmet emotional and cultural needs and limited access to continuous psychological support (Bayuo and Wong, 2021; Kairuz et al., 2023; Lernevall et al., 2021).

Parental challenges frequently persist, and may intensify, during the transition from hospital to home (Egberts et al., 2020). At home, parents assume the role of primary carers, responsible for wound care, rehabilitation, and emotional support. This phase is frequently characterized by insecurity and exhaustion, particularly when discharge preparation and follow-up are insufficient (Lernevall et al., 2021; Wickens et al., 2024). Community-based strategies, such as structured resilience programs, have shown promise in reducing distress and enhancing coping (Schei et al., 2025).

Despite a growing body of research on pediatric burns, previous reviews have largely focused on specific aspects of parental and family experiences, including psychological consequences, parental support needs, and parental distress (Bakker et al., 2013a; Lernevall et al., 2020). However, a comprehensive mapping that integrates these experiences across the continuum of care, from hospitalization to the return home, remains limited. To address the gap, this study aims to systematically map the available evidence on the experiences of parents and carers of children with burns across the continuum of care, focusing on traumatic impact, coping responses, and continuity-of-care challenges. By adopting this perspective, the review seeks to provide an integrated understanding of how parental experiences, coping strategies, family adjustment, and support needs evolve from hospitalization to the return home. This integrated perspective may inform multidisciplinary family-centered care, including the contribution of pediatric psychology to identifying parental distress and support needs across the hospital-to-home continuum.

### 1.1 Conceptual framework

Pediatric burns constitute a traumatic event for the child and the family, particularly for parents who assume a continuous and central role in care (Santos et al., 2024; Wickens et al., 2024). Evidence shows a complex, multifactorial experience that spans emotional (e.g., shock, fear, guilt, anxiety), cognitive (e.g., risk, uncertainty, control), relational (e.g., couple and sibling dynamics), social (e.g., isolation, stigma), and cultural (e.g., norms, values, spirituality) dimensions (Atapattu et al., 2024; Bayuo and Wong, 2021; Coombes et al., 2020). These dimensions interact and evolve across the care trajectory, from admission to discharge and social reintegration, requiring repeated reorganizations of family life and coping (Huo et al., 2024; Shoghi et al., 2019).

In addition to experiencing an emotional impact, parents progressively undertake technical and clinical tasks (e.g., wound care, exercises, pain management, and emotional containment), that were traditionally embedded in professional practice but have been increasingly transferred to families after discharge (Thompson et al., 2022; Townsend et al., 2023). This overlap of practical and emotional demands can heighten insecurity and exhaustion, whereas empowerment and capacity-building strategies are associated with greater parental confidence and reduced stress (Chen et al., 2022; Cioga et al., 2024; Egberts et al., 2019; Mohammadzadeh et al., 2023). To guide the synthesis and interpretation of the findings, we adopted an integrative framework combining three complementary perspectives: the Transactional Model of Stress and Coping (Lazarus and Folkman, 1984), Bronfenbrenner’s Bioecological Model (Bronfenbrenner and Morris, 2006), and Family-Centered Care (Coombes et al., 2020; Kuo et al., 2012), which is closely aligned with the care-partnership concept in pediatric nursing (Casey, 1995).

The Transactional Model of Stress and Coping (Lazarus and Folkman, 1984) explains how parents appraise burn-related demands and mobilize coping strategies that influence adjustment. Within pediatric burn care, this framework supports the interpretation of adaptive and maladaptive coping responses and highlights the importance of multidisciplinary interventions that promote parental resilience and empowerment (Atapattu et al., 2024; Coombes et al., 2020; Foster et al., 2017; Haag and Landolt, 2017; Shaygan et al., 2025; Shoghi et al., 2023).

Complementing this perspective, Bronfenbrenner’s Bioecological Model (Bronfenbrenner and Morris, 2006) situates parental adjustment within interacting family, healthcare, community, and sociocultural systems. It emphasizes that recovery depends not only on injury severity but also on organizational, cultural, and structural factors, supporting the need for coordinated interventions across the continuum of care (Bayuo and Wong, 2021; Townsend et al., 2023; Van Niekerk et al., 2020).

In parallel, Family-Centered Care (FCC) has become foundational in pediatric nursing. It recognizes the family as the unit of care and promotes values of dignity, respect, shared information, collaboration, and participation in decision-making (Kuo et al., 2012). The care-partnership perspective (Casey, 1995) views parents as indispensable partners in decision-making, discharge preparation, continuity of care, and empowerment (Mobayen et al., 2022; Thompson et al., 2022; Townsend et al., 2023).

Together, these complementary perspectives help interpret the thematic findings by integrating cognitive and emotional processes, ecological influences, and family partnership in care. These theoretical foundations informed the synthesis and interpretation of the evidence and supported the discussion of implications for pediatric nursing practice and coordinated multidisciplinary pediatric burn care.

### 1.2 Study purpose

This scoping review aims to systematically map the evidence on (a) the traumatic impact of pediatric burns on parents and family functioning, (b) the coping strategies mobilized by families, and (c) the challenges related to discharge preparation and home care, thereby informing family-centered nursing practice and coordinated multidisciplinary care across the pediatric burn care continuum.

## 2 Materials and methods

This scoping review was designed in accordance with a previously published protocol (Cioga et al., 2025) and was prospectively registered on the Open Science Framework platform.^1^ Thus, this study followed the methodology proposed by the Joanna Briggs Institute (JBI) for scoping reviews, aligned with the PRISMA-ScR (Preferred Reporting Items for Systematic Reviews and Meta-Analyses—Scoping Reviews) extension (Pollock et al., 2024; Tricco et al., 2018). The methodological approach is structured based on the model by Arksey and O’Malley (2005), later improved by Levac et al. (2010), covering the following steps: (1) identification of the research question(s); (2) identification of relevant studies; (3) selection of studies; (4) data extraction and mapping; (5) synthesis and presentation of results.

### 2.1 Review questions

The questions were developed to reflect the objectives of the review and to guide the mapping of the available evidence (Cioga et al., 2025). Parental experiences were defined as encompassing the emotional, caregiving, coping, family, and support-related experiences reported by parents throughout the hospital-to-home continuum following a child’s burn injury.

- What experiences are reported by parents of children who are hospitalized with burns?
- How does the hospitalization process of a child with burns affect parental and family dynamics?
- What coping strategies do parents and families use to care for children hospitalized with burns?
- What are the main challenges faced by parents of children with burns when preparing for the transition from hospital to home?

### 2.2 Inclusion criteria

To define the inclusion and exclusion criteria, the PCC (Participants–Concept–Context) model was adopted, as described in the protocol (Cioga et al., 2025), in accordance with the methodological recommendations of the Joanna Briggs Institute (Peters et al., 2020a,b), as shown in Table 1.

**Table 1:** Inclusion and exclusion criteria.

PCC methodology	Inclusion criteria	Exclusion criteria
Participants	Studies involving children (0–18 years) with accidental burns (hospitalized or outpatient), with no restrictions on the type or duration of the injury, and their parents or legal guardians (biological, adoptive, step-parents, step-mothers, or other formal carers) (UNICEF, 2020). Various family compositions are included, such as single-parent families and cases where parents do not cohabit. No sociodemographic restrictions are applied to carers.	Studies involving non-accidental pediatric burns; studies whose target population does not fall within the pediatric age range (0–18 years) at the time of the burn; studies that do not include parents or carers as participants.
Concept	Studies exploring the psychological and emotional impact associated with pediatric burns and hospitalization on parenting and family dynamics. Parenting is understood as the process that ensures the health and safety of the child and promotes their physical, emotional, social, spiritual, and cognitive development (Jabbari et al., 2024). Family dynamics refer to patterns of interaction, roles, and relationships among family members, influenced by various contextual factors (Azevêdo and Crepaldi, 2019; Jabbari et al., 2024). Psychological and emotional impact, in this context, encompasses distress and, where reported, traumatic stress responses experienced by children and carers following the burn and during hospitalization (De Young et al., 2014; Wickens et al., 2024; Woolard et al., 2021). Also included are studies analyzing the effects of this trauma on parental psychological wellbeing, caregiving practices, family relationships, and coping strategies.	Studies that do not address the experience of parents, guardians, or legal carers, or that do not examine aspects of family dynamics in the context of pediatric burns; studies focused exclusively on biomedical outcomes, disregarding the family experience.
Context	Studies conducted in hospital and home care settings for children with burns and their families, namely in pediatric burn units, intensive care units, general pediatric wards, and specialized rehabilitation centers. Different cultural and socioeconomic contexts are considered to capture the diversity of family experiences. Hospitalization causes significant changes in routines, parental roles, and family relationships, while returning home poses new challenges related to ongoing care, burn sequelae, and the child’s reintegration. Understanding the impact of these transitions is essential to mapping carers’ experiences and informing family-centered care strategies (De Young et al., 2014).	Studies conducted in contexts unrelated to the clinical and family care trajectory of the burned child (e.g., exclusively school-based, legal, or general sociological studies without connection to caregiving or burn-related family experiences).
Types of studies	Quantitative, qualitative, and mixed-methods studies, as well reviews articles and meta-analyses. Theses, dissertations, and institutional reports with empirical data are also included. Studies published in English, Portuguese, and Spanish (languages spoken by the authors) are included, with no time limit.	Sources without original empirical data, such as editorials, letters to the editor, commentaries, or theoretical essays without an investigative basis; documents published in languages other than English, Portuguese, or Spanish.

### 2.3 Search strategy

The literature search proceeded in two stages. In June 2025, we conducted an initial search in MEDLINE/PubMed, Cumulative Index to Nursing and Allied Health Literature [CINAHL] (EBSCOhost), Academic Search Complete, Psychology and Behavioral Sciences Collection (EBSCOhost), APA PsycINFO, Cochrane Library, Scopus, and Web of Science Core Collection (Clarivate). These databases were selected to ensure that a reasonable spread of relevant literature was canvased. In December 2025, we performed an update across all databases to identify newly published records. Strategies—combining controlled vocabularies (e.g., MeSH) and free-text terms with Boolean/proximity operators—were adapted to each source; the full search strings and per-database yields are provided in Supplementary File S1. No date limits were applied; searches were restricted to English, Portuguese, and Spanish. Although the eligibility criteria embraced theses, dissertations, and institutional reports containing empirical data, a systematic search of dedicated gray literature databases was not undertaken. Consequently, the synthesis was based only on studies identified by searching the selected bibliographic databases and by manually screening reference lists of included articles.

### 2.4 Selection of studies

A total of 713 articles were initially identified in the databases. These were exported to the Mendeley software, where 262 duplicates were eliminated. The remaining records were then imported into the Rayyan software platform (Qatar Computing Research Institute, Doha, Qatar) for screening. All abstracts were reviewed, and articles were excluded if they failed to meet all the inclusion criteria. If upon initial screening of the title and/or abstract there was insufficient information for a justified decision, the article was included in the full-text screening phase. Following title and abstract screening, 83 full-text articles were assessed for eligibility, and 63 were included in the final review. A further manual review of all reference lists from included articles was completed. The PRISMA flowchart (Figure 1) shows the total number of records found, the studies selected and discarded, and the reasons for exclusion.

**Figure 1:**
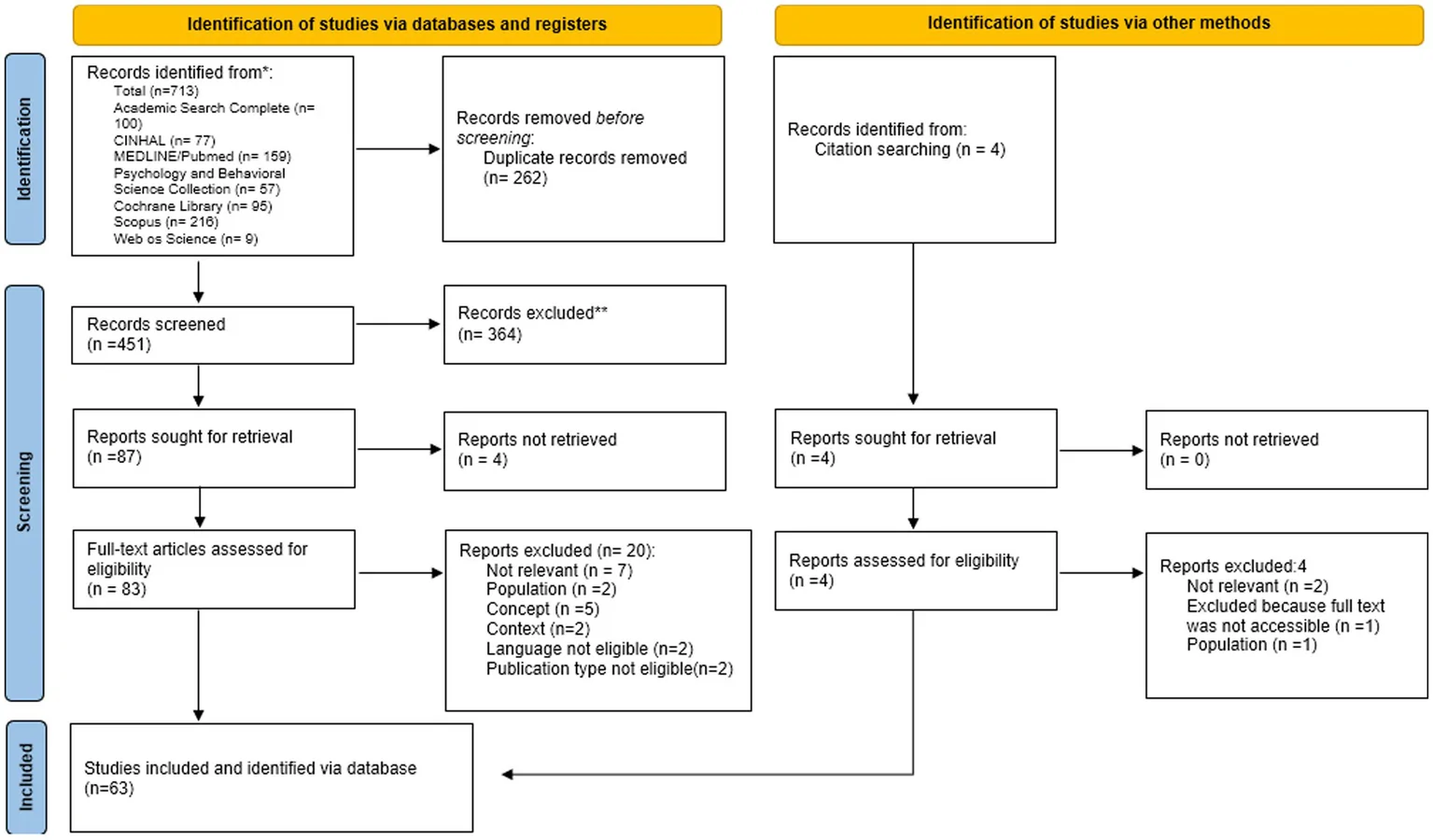
Flowchart of search strategy.

### 2.5 Data extraction

Before initiating the scoping review, we trained reviewers for inclusion/exclusion and coding accuracy using sample articles. The data was collected using an instrument specifically designed by the authors for this purpose, with the aid of Microsoft Excel®. The extraction instrument was refined during training, based on reviewer feedback. In addition to meeting virtually as a group, coauthors and the principal investigator met one-on-one when questions arose about the training process. Data extraction and synthesis were performed independently by two pairs of researchers (E. C., F. V., and C. C., N. C.). Any discrepancies between reviewers were resolved through discussion, with the participation of a third evaluator (J. C. or D. C., depending on the pair involved). For this scoping review, the agreement between dual-blinded reviewers was set at 100% (Peters et al., 2020a).

The data extracted included details about the title, authors, year of publication, country of origin, type of study, study objective, participants; parental experiences; hospital experiences; coping strategies; challenges upon hospital discharge; and main conclusions. For review articles, data were extracted from the review itself (including its objectives, characteristics, synthesized findings, limitations, and recommendations), rather than from the individual primary studies included within the review. Studies published in Portuguese and Spanish were screened, extracted, and analyzed directly by reviewers proficient in these languages; therefore, no translation procedures were required.

### 2.6 Data synthesis and reporting

After extraction, the data were consolidated into a single file and summarized in a characterization table including study context, participants, design, main findings, and conclusions. This descriptive mapping served as the foundation for the subsequent analytical synthesis. A reflexive thematic analysis was then conducted following the approach proposed by Braun and Clarke (2006) and Braun and Clarke (2019), combining deductive coding (informed by the review’s theoretical framework) with inductive coding (derived directly from the data). The analysis followed an iterative and recursive process involving six stages: data familiarization, generation of semantic and latent codes, development of preliminary themes, review of thematic coherence and distinctiveness, refinement and naming of themes, and construction of the final analytical narrative (Braun and Clarke, 2019).

Initial coding was undertaken independently by two reviewers, generating both semantic and latent codes. These codes were subsequently grouped into preliminary categories, which were compared, reviewed, and refined through iterative discussions among the research team. Candidate themes were evaluated for internal coherence, conceptual distinctiveness, and alignment with the review objectives before defining the final themes. This process initially produced a broader set of thematic categories that were progressively consolidated into the three overarching analytical themes and their corresponding subthemes, as presented in the Results section. The refinement process sought to enhance conceptual clarity and consistency while preserving the complexity and breadth of the evidence. To strengthen methodological transparency, Supplementary File S2 presents the progression from preliminary thematic categories to the final analytical themes.

In accordance with the Joanna Briggs Institute (JBI) guidance for scoping reviews (Peters et al., 2020a), no critical appraisal of the included studies was conducted, as the purpose of this review was to map the extent, range, and nature of the available evidence rather than evaluate methodological quality or determine the strength of the evidence. Nevertheless, this should be considered when interpreting the findings, as no conclusions can be drawn regarding the relative robustness of the included studies.

## 3 Results

A total of 63 studies published between 1993 and 2025 were included. Geographically, evidence was mainly concentrated in Europe (44.4%; *n* = 28), followed by North America (17.5%; *n* = 11), Australia (11.1%; *n* = 7), Asia (15.9%; *n* = 10), Africa (7.9%; *n* = 5) and Latin America (3.2%; *n* = 2). The more recent publications encompassed a broader range of geographical settings. Full study details are provided in Supplementary File S3.

Diverse study designs were included: 21 qualitative studies (33.3%), 31 quantitative studies (49.2%), two mixed-methods studies (3.2%), six reviews (9.5%), and three publications classified as “others” (4.8%, including a case study, a protocol paper and a discussion article). The primary studies were distributed across the pediatric burn care continuum: hospital/inpatient = 53.7% (*n* = 29), outpatient/follow-up = 14.8% (*n* = 8), home/community = 13% (*n* = 7), and undefined/unspecified = 18.5% (*n* = 10). Analyses conducted in hospital/inpatient settings primarily addressed the immediate impact of the accident and hospitalization, including acute parental distress, information needs and early role reconfiguration. Outpatient/follow-up studies focused on adherence, rehabilitation and continuity of care. Home/community studies examined the post-discharge transition, family reorganization and social/school reintegration. Samples predominantly included both parents (mother and father) (*n* = 36; 56.9%) or only mothers (*n* = 21; 33.3%), while grandparents were rarely included (*n* = 1; 2.0%), and siblings were seldom direct participants—although they were discussed in some qualitative reports. In 29.4% of studies (*n* = 18), the unit was reported generically (“family”/“carer”), without kinship detail. The predominance of maternal participants reflects caregiving availability but limits comparability and constrains analysis of the role of siblings in family adjustment.

### 3.1 Navigating challenging parental and family dynamics during the pediatric burn recovery

To provide a concise overview of how the 63 included studies contributed to the thematic synthesis, Table 2 summarizes the distribution of studies across the three analytic themes. Although several studies addressed more than one thematic dimension, each study was assigned to the theme most directly aligned with its primary focus. The thematic synthesis identified three conceptually distinct analytic themes. Each theme captures a different dimension of parents’ and families’ experiences following a pediatric burn injury, while together they illustrate the broader process of parental and family adjustment across the continuum of care. The overarching theme “Navigating Challenging Parental and Family Dynamics during the Pediatric Burn Recovery” integrates the three analytic themes. These represent distinct dimensions of parental and family adjustment, capturing the traumatic impact on families, the strategies mobilized to cope, and the challenges encountered in preparing for discharge and maintaining continuity of care.

**Table 2:** Mapping of included studies across analytic themes and subthemes.

Main theme	Analytic themes	Subthemes
Navigating challenging parental and family dynamics during the pediatric burn recovery	Theme 1—traumatic impact of pediatric burns and consequences for family dynamics	Emotional response and psychological overload (Atapattu et al., 2024; Bakker et al., 2013a,b; Bayuo and Wong, 2021; Blakeney et al., 1993; Carvalho et al., 2008; Chen et al., 2022; Cioga et al., 2024; Coombes et al., 2020; De Young et al., 2014; Egberts et al., 2020; Egberts et al., 2019; Egberts et al., 2018; El Hamaoui et al., 2006; Foster et al., 2017; Haag and Landolt, 2017; Heath et al., 2019; Kilburn and Dheansa, 2014; Ledoux et al., 1998; Lernevall et al., 2021; Lernevall et al., 2023; Mazzi et al., 2010; McGarry et al., 2013; Morley et al., 2017; Odar et al., 2013; Oliveira et al., 2015; Öster et al., 2014; Patel et al., 2022; Patterson et al., 1993; Phillips and Rumsey, 2008; Ravindran et al., 2013; Rimmer et al., 2015; Rizzone et al., 1994; Rose et al., 2005; Rossi et al., 2005; Santos et al., 2025; Santos et al., 2024; Schei et al., 2025; Shaygan et al., 2025; Shoghi et al., 2023; Smith et al., 2011; Suurmond et al., 2020; Townsend et al., 2023; Van Niekerk et al., 2020; Wickens et al., 2024; Willebrand and Sveen, 2016a)
		Impact on family functioning and relationships (Atapattu et al., 2024; Bakker et al., 2013a,b; Bayuo and Wong, 2021; Blakeney et al., 1993; Cioga et al., 2024; Coombes et al., 2020; Coy et al., 2019; Egberts et al., 2019; Foster et al., 2017; Heath et al., 2019; Johnson et al., 2016; Khan et al., 2024; Kilburn and Dheansa, 2014; Kornhaber et al., 2018; Ledoux et al., 1998; Lernevall et al., 2023; Mazzi et al., 2010; McGarry et al., 2013; Mohammadzadeh et al., 2023; Morley et al., 2017; Pan et al., 2018; Parrish et al., 2019; Patel et al., 2022; Rangraz Jeddi et al., 2023; Rimmer et al., 2015; Santos et al., 2025; Santos et al., 2024; Schei et al., 2025; Suurmond et al., 2020; Thompson et al., 2022; Townsend et al., 2023; Van Niekerk et al., 2020; Wickens et al., 2024)
		Influence of contextual factors (Atapattu et al., 2024; Bayuo and Wong, 2021; Carvalho et al., 2008; Cioga et al., 2024; Coy et al., 2019; Egberts et al., 2018; Foster et al., 2017; Kairuz et al., 2023; Khan et al., 2024; Kilburn and Dheansa, 2014; Kornhaber et al., 2018; Lernevall et al., 2020; Mazzi et al., 2010; Öster et al., 2014; Pan et al., 2018; Phillips and Rumsey, 2008; Rangraz Jeddi et al., 2023; Ravindran et al., 2013; Rose et al., 2005; Rossi et al., 2005; Santos et al., 2025; Schei et al., 2025; Suurmond et al., 2020; Van Niekerk et al., 2020; Wickens et al., 2024)
	Theme 2—coping strategies of parents and families	Emotion-focused coping (Blakeney et al., 1993; Egberts et al., 2019; Egberts et al., 2018; El Hamaoui et al., 2006; Foster et al., 2017; Haag and Landolt, 2017; Huo et al., 2024; Johnson et al., 2016; Jopke et al., 2017; Lernevall et al., 2020; Lernevall et al., 2021; Morley et al., 2017; Öster et al., 2014; Pan et al., 2018; Ravindran et al., 2013; Santos et al., 2025; Schei et al., 2025; Shaygan et al., 2025; Thompson et al., 2022; Wickens et al., 2024)
		Problem-focused coping (Atapattu et al., 2024; Bakker et al., 2013a; Cioga et al., 2024; Coombes et al., 2020; Haag and Landolt, 2017; Heath et al., 2019; Huo et al., 2024; Johnson et al., 2016; Ledoux et al., 1998; Lernevall et al., 2023; Mazzi et al., 2010; McGarry et al., 2013; Morley et al., 2017; Pan et al., 2018; Rangraz Jeddi et al., 2023; Rimmer et al., 2015; Schei et al., 2025; Shoghi et al., 2023; Smith et al., 2011; Thompson et al., 2022; Tyack and Ziviani, 2003; Willebrand and Sveen, 2016b)
		Meaning-making strategies (Bayuo and Wong, 2021; Cioga et al., 2024; Coombes et al., 2020; Coy et al., 2019; Egberts et al., 2019; Foster et al., 2017; Khan et al., 2024; Kornhaber et al., 2018; Lernevall et al., 2020; Mazzi et al., 2010; Öster et al., 2014; Ravindran et al., 2013; Rose et al., 2005; Suurmond et al., 2020; Van Niekerk et al., 2020; Willebrand and Sveen, 2016a)
		Maladaptive strategies (Bakker et al., 2014; Chen et al., 2022; De Young et al., 2014; Egberts et al., 2019; El Hamaoui et al., 2006; Kornhaber et al., 2018; Öster et al., 2014; Patterson et al., 1993; Phillips and Rumsey, 2008; Rose et al., 2005; Rossi et al., 2005; Santos et al., 2025; Schei et al., 2025; Suurmond et al., 2020; Willebrand and Sveen, 2016a)
	Theme 3—preparation for discharge and continuity of care	Living transitions for home care (Carvalho et al., 2008; Cioga et al., 2024; Coy et al., 2019; De Young et al., 2014; Egberts et al., 2019; El Hamaoui et al., 2006; Lernevall et al., 2023; Mazzi et al., 2010; Mohammadzadeh et al., 2023; Morley et al., 2017; Öster et al., 2014; Pan et al., 2018; Phillips and Rumsey, 2008; Rangraz Jeddi et al., 2023; Rimmer et al., 2015; Rizzone et al., 1994; Santos et al., 2024; Smith et al., 2011; Thompson et al., 2022; Tyack and Ziviani, 2003; Van Niekerk et al., 2020; Wickens et al., 2024; Willebrand and Sveen, 2016b)
		Challenges in discharge preparation and education (Atapattu et al., 2024; Coombes et al., 2020; Egberts et al., 2019; Egberts et al., 2018; Johnson et al., 2016; Khan et al., 2024; Lernevall et al., 2021; Morley et al., 2017; Öster et al., 2014; Özer and Vural, 2020; Phillips and Rumsey, 2008; Santos et al., 2025; Schei et al., 2025; Smith et al., 2011; Thompson et al., 2022; Tyack and Ziviani, 2003; Van Niekerk et al., 2020; Wickens et al., 2024)
		Post-discharge continuity, safety, and integration (Atapattu et al., 2024; Bayuo and Wong, 2021; Cioga et al., 2024; Heath et al., 2019; Kairuz et al., 2023; Khan et al., 2024; Öster et al., 2014; Patel et al., 2022; Phillips and Rumsey, 2008; Rose et al., 2005; Santos et al., 2025; Schei et al., 2025; Shoghi et al., 2019; Townsend et al., 2023; Van Niekerk et al., 2020; Wickens et al., 2024)

The included studies consistently described emotional and practical challenges experienced by parents and families following pediatric burn injury. During the acute phase, parents reported emotional distress, prognostic uncertainty, fear, guilt, and disruptions to family organization and daily life (Carvalho et al., 2008; McGarry et al., 2013; Ravindran et al., 2013). As recovery progressed, families described adapting to new caregiving responsibilities, reorganizing routines, renegotiating family roles, and attempting to restore a sense of normality within the household (Lernevall et al., 2023). Across the care trajectory, studies also described a range of resources available to parents and families, including clear and empathic communication from healthcare professionals (Mazzi et al., 2010; Pan et al., 2018), access to psychological support (El Hamaoui et al., 2006), family and community support networks (Foster et al., 2013; Van Niekerk et al., 2020), empowerment and education initiatives (Johnson et al., 2016; Mohammadzadeh et al., 2023), and structured follow-up (Öster et al., 2014; Willebrand and Sveen, 2016b).

### 3.2 Theme 1—traumatic impact of pediatric burns and consequences for family dynamics

Pediatric burns have a lasting impact on parents and families, from hospital admission through the post-discharge phase. During hospitalization, parents commonly experience shock, fear, helplessness, guilt, and acute stress in response to the burn event. Following discharge, these emotional reactions may persist alongside ongoing psychological symptoms, including anxiety, depressive symptoms, and, in some studies, PTSS. These individual impacts occur alongside family reorganization (role shifts, sibling demands, marital strain, and reduced cohesion, often with greater maternal burden). Stigma, cultural or spiritual pressures, and socioeconomic conditions were also reported in relation to these experiences. Findings are presented according to the stage in the care trajectory (hospitalization and post-discharge) to describe the impact of pediatric burns on parents and family functioning across the continuum of care.

### 3.3 Emotional response and psychological overload

The initial emotional impact is substantial. Parents describe disbelief, panic, acute stress, and intrusive memories. The intense psychological burden is described in relation to prognostic and treatment uncertainty and is expressed as anxiety, insomnia, fatigue, persistent tension, and emotional overload (Carvalho et al., 2008; Haag and Landolt, 2017; Ledoux et al., 1998; McGarry et al., 2013; Oliveira et al., 2015; Ravindran et al., 2013). Recurrent parental guilt, often accompanied by shame and self-recrimination, is reinforced by moral interpretations and social expectations (Bayuo and Wong, 2021; El Hamaoui et al., 2006; Lernevall et al., 2021; Patterson et al., 1993; Rizzone et al., 1994; Suurmond et al., 2020), while challenges to parental identity are also described (Ravindran et al., 2013). Anxiety is frequently reported, often together with insomnia, fatigue, and tension (Bakker et al., 2013a; Carvalho et al., 2008; Foster et al., 2017; Morley et al., 2017; Phillips and Rumsey, 2008; Willebrand and Sveen, 2016a), and is highest before changing of dressings (Phillips and Rumsey, 2008; Rimmer et al., 2015; Smith et al., 2011). Some quantitative studies also report early PTSS (Lernevall et al., 2023; Mazzi et al., 2010). Longitudinally, distress is highest at admission but tends to decline (Bakker et al., 2013b; Egberts et al., 2020; Santos et al., 2025).

After discharge, initial feelings often persist or intensify, such as ongoing fear, anxiety, insecurity, and related symptoms (such as nightmares, flashbacks, hypervigilance) that anchor parents to the moment of the burn (Atapattu et al., 2024; Bakker et al., 2013b; Egberts et al., 2020; Lernevall et al., 2023; Öster et al., 2014; Patel et al., 2022; Phillips and Rumsey, 2008; Wickens et al., 2024). Observational data indicates a notable prevalence of PTSS (Odar et al., 2013). Qualitative evidence highlights delayed emotional “collapse,” sometimes with reduced work functioning (Schei et al., 2025). Although parental distress decreases, family functioning worsens and fully mediates the link between distress and burn-scar quality (Santos et al., 2025). Maternal guilt and shame are frequently described, and some quantitative studies report associations with depressive symptoms (Egberts et al., 2018; Townsend et al., 2023). Stigma and community judgment are reported alongside social isolation, with gendered patterns favoring maternal self-blame (Egberts et al., 2020; Coombes et al., 2020; Heath et al., 2019). Anxiety often persists or worsens and is related to poorer family functioning and quality of life (Atapattu et al., 2024; Bakker et al., 2013b; Egberts et al., 2019; Egberts et al., 2018; Öster et al., 2014; Phillips and Rumsey, 2008; Rose et al., 2005; Wickens et al., 2024). Lack of marital or social support exacerbates parental burden (Blakeney et al., 1993; Phillips and Rumsey, 2008; Rose et al., 2005; Wickens et al., 2024). Technical home-care demands (analgesia, rehabilitation, dressings, and appointments) provoke fatigue and disrupt routines (Cioga et al., 2024; Heath et al., 2019; Shoghi et al., 2023; Van Niekerk et al., 2020), and obsessive-compulsive symptoms are reported (Chen et al., 2022). Parents actively redefine routines to ensure safety at home (Kilburn and Dheansa, 2014).

### 3.4 Impact on family functioning and relationships

During hospitalization, families undergo rapid reconfiguration of roles and identity, with accumulation of responsibilities, emotional restraint and insecurity regarding care (Bayuo and Wong, 2021; Egberts et al., 2019; Heath et al., 2019; McGarry et al., 2013; Morley et al., 2017; Oliveira et al., 2015; Schei et al., 2025; Suurmond et al., 2020; Thompson et al., 2022; Wickens et al., 2024). Professionals sometimes underestimate parental overload (Lernevall et al., 2021; Schei et al., 2025; Smith et al., 2011). Parental emotional states and the family environment are related to the child’s behavioral adjustment (Ledoux et al., 1998; Santos et al., 2025). Managing siblings’ needs adds complexity (Foster et al., 2017; Santos et al., 2024; Schei et al., 2025). Marital conflict and parent–child tension (including blaming the child) weaken cohesion (Bakker et al., 2013a,b; Bayuo and Wong, 2021; Blakeney et al., 1993; Coy et al., 2019; Egberts et al., 2019; Heath et al., 2019; Johnson et al., 2016; Khan et al., 2024; Kornhaber et al., 2018; McGarry et al., 2015; Patterson et al., 1993; Ravindran et al., 2013; Shoghi et al., 2023). Routines and tasks are reorganized, often increasing siblings’ involvement and emotional or logistical load (Bayuo and Wong, 2021; Carvalho et al., 2008; Heath et al., 2019; Huo et al., 2024; Kornhaber et al., 2018; McGarry et al., 2013; Morley et al., 2017; Parrish et al., 2019; Patel et al., 2022; Rimmer et al., 2015; Schei et al., 2025; Suurmond et al., 2020; Thompson et al., 2022). Family cohesion and organization are related to better adjustment (Ledoux et al., 1998).

After discharge, uncertainty often persists or intensifies in everyday family life, leading to parenting difficulties and an almost exclusive focus on the burned child, with reduced availability for siblings (Atapattu et al., 2024; Bakker et al., 2013a; Cioga et al., 2024; Santos et al., 2025; Schei et al., 2025; Townsend et al., 2023; Wickens et al., 2024). Structured empowerment programs are associated with gains in confidence and competence (Mohammadzadeh et al., 2023; Thompson et al., 2022). Marital conflicts and socioeconomic pressures persist/worsen with gendered patterns and erosion of family cohesion (Cioga et al., 2024; Coy et al., 2019; Huo et al., 2024; Lernevall et al., 2020; Lernevall et al., 2023; Morley et al., 2017; Rangraz Jeddi et al., 2023; Santos et al., 2025; Thompson et al., 2022; Townsend et al., 2023; Wickens et al., 2024). Siblings often feel jealous and excluded by their parents, who they perceive as overprotecting the injured child (Kilburn and Dheansa, 2014; Schei et al., 2025; Van Niekerk et al., 2020). Prolonged role redefinitions and adjustments to domestic and school routines are reported alongside continued demands on parents and siblings (Atapattu et al., 2024; Cioga et al., 2024; Coombes et al., 2020; Heath et al., 2019; Parrish et al., 2019; Rangraz Jeddi et al., 2023; Santos et al., 2025; Santos et al., 2024; Schei et al., 2025; Townsend et al., 2023; Van Niekerk et al., 2020). In some contexts, overprotection is related to long-term adjustment difficulties (Willebrand and Sveen, 2016a). In response to the lasting risks, parents actively redefine routines to promote safety in the home (Kilburn and Dheansa, 2014).

### 3.5 Influence of contextual factors

Experiences during admission are shaped by context. Stigma related to visible scarring and moral interpretations amplify guilt and shame (Kornhaber et al., 2018; Phillips and Rumsey, 2008; Ravindran et al., 2013; Schei et al., 2025). Cultural and religious or spiritual pressures intensify distress (Bayuo and Wong, 2021; Carvalho et al., 2008; Kornhaber et al., 2018; Lernevall et al., 2020; Suurmond et al., 2020). In resource-constrained settings, psychological overload co-occurs with socioeconomic strain and spiritual distress, and sometimes with intra-family conflict or violence (Khan et al., 2024). Access barriers (income loss, costs, and structural obstacles) are particularly detrimental for families with linguistic or psychosocial vulnerabilities (Bayuo and Wong, 2021; Egberts et al., 2018; Khan et al., 2024; Kilburn and Dheansa, 2014).

After discharge, stigma often persists or worsens, fostering isolation, fear of exposure, and social withdrawal (Cioga et al., 2024; Coy et al., 2019; Öster et al., 2014; Rose et al., 2005; Rossi et al., 2005; Schei et al., 2025). Emotional burden remains high, with greater maternal withdrawal when local support is scarce (Mazzi et al., 2010; Schei et al., 2025; Wickens et al., 2024). Families from cultural or linguistic minorities experience greater vulnerability and disparities (Atapattu et al., 2024; Townsend et al., 2023). Supportive school and community environments facilitate reintegration and reduce experiences of exclusion and bullying (Foster et al., 2017; Van Niekerk et al., 2020). Persistent economic challenges impair adaptation and the quality of home care (Kairuz et al., 2023; Khan et al., 2024; Schei et al., 2025; Wickens et al., 2024), as financial burden endangers parental mental health and child wellbeing (Rangraz Jeddi et al., 2023; Santos et al., 2024). Co-ordinated school re-entry plans and access to peer and digital support are associated with reduced avoidance and smoother normalization, whereas persistent financial or structural barriers prolong strain (Heath et al., 2019; Kairuz et al., 2023; Schei et al., 2025; Van Niekerk et al., 2020).

### 3.6 Theme 2—coping strategies of parents and families

Following pediatric burn injury, parents and families use different coping strategies across hospital and post-discharge contexts. In this review, findings were organized into four axes: (i) emotion-focused coping (e.g., regulation, acceptance/reframing, positive reinterpretation); (ii) problem-focused coping (e.g., active involvement in care, seeking information, reorganizing routines); (iii) maladaptive strategies (e.g., avoidance, denial, hypervigilance, self-blame); and (iv) meaning-making strategies (Park, 2013). Meaning-making included: (a) religious/spiritual coping, where faith and spiritual practices offered comfort and hope, although negative interpretations (e.g., divine punishment) were associated with greater distress; (b) purpose-oriented reframing, centered on the child’s recovery and a reordering of priorities; and (c) family-based meaning-making, in which the family unit served as a source of connection and shared understanding. While recognizing that some coping patterns may hinder adjustment, the findings illustrate how parents and families mobilize different coping strategies across the care trajectory to respond to the emotional, practical and relational challenges associated with pediatric burns.

### 3.7 Emotion-focused coping

In the hospital studies, the use of emotional regulation (empathy, humor, resilience) and, at times, family emotional interdependence was also described as a protective response, although it could also contribute toward emotional overload (Egberts et al., 2019; Egberts et al., 2018; Foster et al., 2017; Schei et al., 2025). Emotional restraint or suppression may impede the fulfillment of the therapeutic caregiving role (Schei et al., 2025; Thompson et al., 2022). Programs labeled as resilience training can improve regulation and acute-stress management (Haag and Landolt, 2017; Shaygan et al., 2025). Clear, empathic communication within a trusting relationship (Pan et al., 2018) and access to psychological support, including individual or group therapy (El Hamaoui et al., 2006), are described together with earlier emotional re-organization during admission (El Hamaoui et al., 2006; Haag and Landolt, 2017; Pan et al., 2018; Schei et al., 2025; Shaygan et al., 2025). Professional psychosocial support is reported as important for maintaining the parent–child bond (Blakeney et al., 1993).

Following discharge, emotional adjustment continues to evolve. Proximity to professionals remains a source of security and shared humor provides relief (Johnson et al., 2016; Lernevall et al., 2020; Lernevall et al., 2023). In the presence of persistent hypervigilance and anxiety, some parents report mindfulness and self-care (Wickens et al., 2024). Parents described suppressing or accepting suffering alongside gradual acceptance of the parental role and strengthened marital coping (Morley et al., 2017; Öster et al., 2014; Ravindran et al., 2013; Rose et al., 2005). Quantitative studies further indicated that social support moderated the association between perceived parental stress and anxiety, depression, and sleep quality (Huo et al., 2024). Continued psychosocial support is associated with attenuation of guilt and depressive symptoms (Egberts et al., 2019), while psychosocial interventions, structured empowerment programs and support networks are associated with higher parental resilience and post-traumatic growth, confidence, self-esteem and adaptation (Atapattu et al., 2024; Cioga et al., 2025; De Young et al., 2014; Jopke et al., 2017; Mohammadzadeh et al., 2023).

According to the studies included, emotion-focused coping was described as evolving over time, with continued psychosocial support associated with greater emotional adaptation (Atapattu et al., 2024; Santos et al., 2025; Schei et al., 2025).

### 3.8 Problem-focused coping

Within hospital care, many parents adopt action-centered coping, becoming co-producers of care by participating in dressing changes, rehabilitation exercises and daily ward routines; this practice is associated with a restored sense of control and reduced helplessness (Coombes et al., 2020; Ledoux et al., 1998; McGarry et al., 2013; Morley et al., 2017; Rimmer et al., 2015; Tyack and Ziviani, 2003). Parents adopt procedural strategies, such as remaining present during procedures, using distraction techniques, and speaking in familiar language to alleviate the child’s distress and reinforce the parental role (Smith et al., 2011). Digital tools based on cognitive-behavioral principles, together with peer testimonials, have been implemented in some burn units (Heath et al., 2019). Parents are progressively involved in care through supervised rehearsals before the first independent dressing or care episode and by observing and gradually participating in bedside routines. Supervised practice and explicit validation of competence during rehabilitation is associated with greater parental self-efficacy, smoother coordination with staff, increased perceived control and readiness for autonomous care (Johnson et al., 2016; Rimmer et al., 2015; Schei et al., 2025; Thompson et al., 2022; Tyack and Ziviani, 2003). Parents consistently value empathy, transparency and inclusion in routines (Johnson et al., 2016; Santos et al., 2024). Positive evaluations of the care team and trust in the treatment are associated with greater readiness to provide autonomous care (Mazzi et al., 2010).

Following discharge, parents consolidate the practical skills acquired during hospitalization by applying techniques learned from healthcare professionals during wound care, rehabilitation and follow-up, and by maintaining ongoing communication with the healthcare team to validate care practices and reinforce confidence in home care (De Young et al., 2014; Lernevall et al., 2020; Townsend et al., 2023). This process is further strengthened through parental empowerment programs, peer support initiatives such as Family Burn Camp, and active information-seeking supported by educational resources and ongoing communication with healthcare professionals and schools (Atapattu et al., 2024; Cioga et al., 2024; Rangraz Jeddi et al., 2023; Santos et al., 2024; Schei et al., 2025). Structured follow-up was described in relation to the management of emerging challenges, progressive reorganization of family routines (including role redefinition), and the child’s school and social reintegration. Adaptations to the home environment to promote safety and prevent further burn injuries were also reported (Coy et al., 2019; Foster et al., 2017; Ledoux et al., 1998; Schei et al., 2025).

Although parents generally report progressive improvements in coping over time, some studies also describe persistent difficulties in family functioning during the post-discharge period (Rossi et al., 2005).

### 3.9 Meaning-making strategies

Spirituality and faith are frequently described as primary resources early in the journey of care, as they help manage acute suffering, by offering comfort, hope and a symbolic frame. Acknowledgement of spiritual needs in family-centered, culturally-sensitive care is linked to reassurance and trust in staff (Foster et al., 2017; Lernevall et al., 2020; Mazzi et al., 2010; Ravindran et al., 2013; Rose et al., 2005; Suurmond et al., 2020). In immigrant or culturally diverse families, rituals and religious practices are protective, structuring experiences that mobilize support (Lernevall et al., 2020; Suurmond et al., 2020). In resource-constrained contexts, cultural and religious pressures, together with family and community judgments surrounding the burn event, were associated with increased parental guilt, shame, and emotional distress, and in some cases with intra-family conflict or violence (Bayuo and Wong, 2021; Khan et al., 2024).

Over subsequent months, spirituality is often described as a structuring force: trust in divine protection, meaning-making, and support from faith or community networks occur alongside adaptive reappraisal and social reintegration, frequently articulated with peer support (Bayuo and Wong, 2021; Cioga et al., 2024; Coombes et al., 2020; Coy et al., 2019; Kornhaber et al., 2018; Öster et al., 2014; Rose et al., 2005). School and community awareness, combined with links to faith communities, facilitates smoother reintegration (Foster et al., 2017; Van Niekerk et al., 2020). However, some studies also describe negative spiritual interpretations associated with shame, avoidance and delayed help-seeking (Egberts et al., 2019; Khan et al., 2024; Willebrand and Sveen, 2016a).

### 3.10 Maladaptive strategies

During hospitalization, to manage the immediate emotional burden, parental avoidance takes the form of partial denial and defensive minimization of the burn event or its severity, sometimes accompanied by isolation and refusal to acknowledge its consequences (Blakeney et al., 1993; El Hamaoui et al., 2006). Medium-term reports link avoidance with greater psychological distress and suffering (Chen et al., 2022; Patterson et al., 1993). Role strain and pressure to “hold it together” as therapeutic carers are also related with emotional suppression or avoidance (Schei et al., 2025).

In the period following hospitalization, parental avoidance is described as social withdrawal and escape from situations reminiscent of the accident; public exposure is avoided due to scar-related stigma and fear of judgment, burn marks are hidden, and community contact is limited (Coy et al., 2019; Öster et al., 2014; Phillips and Rumsey, 2008; Rossi et al., 2005; Suurmond et al., 2020). Many studies report hypervigilance or overprotection (constant monitoring, restriction of activities and risk avoidance), together with parental PTSS and, in some cases, isolation and emotional suppression (Bakker et al., 2014; Wickens et al., 2024; Willebrand and Sveen, 2016a). Self-blame and self-accusation are also reported. Blame directed toward the child is associated with relational strain, whereas internalized guilt and shame are associated with a higher risk of PTSS and depression. Culturally shaped passive or resigned coping patterns are also described (Blakeney et al., 1993; De Young et al., 2014; Egberts et al., 2019; Kornhaber et al., 2018; Rose et al., 2005). Maladaptive coping is reported throughout the recovery trajectory, particularly in contexts of stigma and limited informal support (Rossi et al., 2005; Wickens et al., 2024). Longitudinal evidence suggests improvement in parental distress and coping, but worsening family functioning after discharge (Santos et al., 2025).

### 3.11 Theme 3—preparation for discharge and continuity of care

The hospital–home transition is associated with anticipatory anxiety, insecurity and emotional distress, although this varies with available information and practical training. Across sources, phased education, bedside rehearsal with competence validation, and accessible follow-up are related to smoother day-to-day management. Generic instruction, limited rehearsal and restricted access are described as contributing to difficulties in establishing home care routines. Parental vulnerability is frequently described. Findings are organized in three subthemes: (i) Living transitions for home care, (ii) Challenges in discharge preparation and education, and (iii) Post-discharge continuity, safety, and integration. Together, these findings describe how families experience discharge preparation and continuity of care while adapting to home care throughout the transition from hospital to home.

### 3.12 Living transitions for home care

In the context of hospitalization, parents describe anticipatory anxiety (around dressings, analgesia, splints, physiotherapy and scar care), with peaks before dressings (Phillips and Rumsey, 2008; Smith et al., 2011), together with feelings of helplessness, sadness and fatigue (Carvalho et al., 2008; Rimmer et al., 2015). Parents describe emotional suppression while attempting to fulfill their therapeutic caregiving role (Lernevall et al., 2021). Quantitative reports describe associations between obsessive-compulsive symptoms, parental anxiety and adherence difficulties (Chen et al., 2022). Family functioning is relevant to adherence (Tyack and Ziviani, 2003) and acute parental stress is associated with later post-traumatic responses (De Young et al., 2014). Maternal vulnerability (stress/depression) tends to increase (El Hamaoui et al., 2006; Rizzone et al., 1994). Perceived quality of the therapeutic relationship, empathy, transparency and inclusion are linked to lower procedural anxiety and greater readiness; trust in treatment is associated with security (Mazzi et al., 2010; Santos et al., 2024). Good communication and a trusting therapeutic relationship are associated with lower procedural anxiety and greater readiness for discharge (Pan et al., 2018). Phased, plain-language briefings, bedside rehearsal with explicit validation, and take-home written/visual materials tend to lower procedural anxiety and foster a higher perceived readiness for discharge and home care (Johnson et al., 2016; Morley et al., 2017; Pan et al., 2018; Phillips and Rumsey, 2008; Rimmer et al., 2015; Smith et al., 2011; Tyack and Ziviani, 2003).

After discharge, these challenges intensify and returning home is experienced with insecurity and a need for continued professional support (Öster et al., 2014), compounded by guilt, anxiety and intrusive memories (Shoghi et al., 2019). PTSS and avoidance tend to hinder adherence (Willebrand and Sveen, 2016b). In the initial weeks, distress is centered on technical tasks (Egberts et al., 2019). Maternal vulnerability persists, particularly where parental PTSS co-occurs with severe burns (El Hamaoui et al., 2006; Rizzone et al., 1994). Day-to-day management and adherence can be fostered through early structured contacts (telephone/tele-follow-up in week 1; pre-booked appointments), parent-initiated competence reinforcement (tele-check-ins) and written/visual plus peer/digital resources (Atapattu et al., 2024; Cioga et al., 2024; Lernevall et al., 2023; Öster et al., 2014; Rangraz Jeddi et al., 2023; Santos et al., 2024). Family empowerment programs improve parental confidence and competence; mothers describe greater security with institutional support (Mazzi et al., 2010; Mohammadzadeh et al., 2023). Normalization is associated with re-engagement with family/school routines (Coy et al., 2019; Van Niekerk et al., 2020).

### 3.13 Challenges in discharge preparation and education

During the hospital stay, discharge preparation is frequently described as insufficient (generic instruction, limited supervised practice, and absence of structured plans) (Lernevall et al., 2021). The need for clear information and practical training is mentioned often (Coombes et al., 2020; Smith et al., 2011), as well as insecurity with early discharges and risks of emotional repression (Öster et al., 2014; Thompson et al., 2022). Gaps in pain management and physiotherapy are persistent (Morley et al., 2017; Özer and Vural, 2020). Structured education—phased briefings, supervised rehearsal with validation and take-home materials—are associated with higher readiness (Egberts et al., 2018; Schei et al., 2025; Smith et al., 2011; Tyack and Ziviani, 2003; Van Niekerk et al., 2020). Financial/logistical barriers limit participation in bedside education in resource-constrained settings (Khan et al., 2024).

During the first weeks following hospitalization, there are preparation gaps associated with prolonged insecurity and emotional distress, isolation, uncertainty in resource use and insufficient information (Chen et al., 2022; Johnson et al., 2016; Phillips and Rumsey, 2008). Absence of structured plans is associated with heightened vulnerability and delayed “emotional breakdown” when support is scarce (Santos et al., 2025; Schei et al., 2025; Van Niekerk et al., 2020). Persistent PTSS months after discharge is reported in several studies (Atapattu et al., 2024; Wickens et al., 2024), alongside earlier reports of anticipatory stress during hospitalization (Atapattu et al., 2024; Wickens et al., 2024). Normalization is once again related with re-engagement in family and school routines (Coy et al., 2019; Van Niekerk et al., 2020).

### 3.14 Post-discharge continuity, safety, and integration

During the return-home phase, safety and integration are associated with coordinated, family-oriented follow-up, linking the hospital, primary care, schools and communities. School/community support was reported as important for normalization; digital strategies and peer groups (including Family Burn Camp) reduce isolation and strengthen confidence (Cioga et al., 2024; Heath et al., 2019; Schei et al., 2025; Van Niekerk et al., 2020). Cultural/linguistic minority families report greater vulnerability and disparities, while family and community networks are described as central resources (Bayuo and Wong, 2021; Kairuz et al., 2023; Khan et al., 2024; Townsend et al., 2023). Persistent PTSS and ongoing feelings of guilt, shame and self-blame are reported several months after discharge (Atapattu et al., 2024; Kornhaber et al., 2018; Patel et al., 2022; Phillips and Rumsey, 2008; Rose et al., 2005; Wickens et al., 2024). Accessible professionals and reliable contact channels (named point-of-contact; early tele-follow-up) are associated with smoother day-to-day normalization and timely help-seeking (Cioga et al., 2024; Heath et al., 2019; Schei et al., 2025; Wickens et al., 2024). Reports describe decreases in parental distress post-discharge, but worsening family functioning; family functioning seems to fully mediate the association between parental distress and burn-scar quality (Santos et al., 2025). Limited access to professional and social support contributes to ongoing parental strain and persistent stress (Atapattu et al., 2024; Heath et al., 2020; Kairuz et al., 2023; Khan et al., 2024; Öster et al., 2014; Rose et al., 2005; Townsend et al., 2023; Van Niekerk et al., 2020; Wickens et al., 2024).

## 4 Discussion

The aim of this scoping review was to map the parental trajectory in the context of pediatric burns and to identify the resources perceived as shaping family adjustment across the hospital–home continuum. Collectively, the findings suggest that parents’ experiences evolve across the continuum of care, with emotional responses, coping strategies, and support needs changing from hospitalization to the return home. This highlights that parental adjustment is an ongoing process, shaped by changing individual, family, and healthcare-related demands throughout the child’s care trajectory. Across these levels, communication with healthcare professionals, psychosocial support, family and community networks, parental empowerment and education, and continuity of follow-up emerge as important resources throughout the adjustment process. This interpretation draws on three complementary conceptual frameworks: the Transactional Model of Stress and Coping (Lazarus and Folkman, 1984), which elucidates how threat and control appraisals guide coping; the Bioecological Model of Development (Bronfenbrenner and Morris, 2006), which situates adjustment within layers of ecological influence; and Family-Centered Care (Kuo et al., 2012), associated with a care-partnership perspective (Casey, 1995), which emphasizes co-production, predictability, and validation of parental competence.

Hospitalization represents the most disruptive phase of the parental adjustment trajectory, during which intense emotional distress, uncertainty, and family reorganization are particularly evident (Egberts et al., 2018; Foster et al., 2017; Phillips and Rumsey, 2008; Wickens et al., 2024). Painful procedures, such as dressing changes and rehabilitation, further reinforce this disruption and may undermine parental confidence in caring for their child after discharge (Atapattu et al., 2024; Öster et al., 2014; Santos et al., 2024; Schei et al., 2025). These responses parallel evidence from other areas of pediatric critical illness, where hospitalization is portrayed as a biographical rupture, associated with a loss of autonomy and disruption in family organization (Dewan et al., 2025; Olson et al., 2024). The impact extends to family dynamics, with the burden falling disproportionately on the primary caregiver, increasing conjugal tension and reducing availability for siblings (Coombes et al., 2020; Kilburn and Dheansa, 2014; Van Niekerk et al., 2020). Parental guilt is reinforced by cultural expectations and perceived social judgment (Bayuo and Wong, 2021; Egberts et al., 2019; Kornhaber et al., 2018; Olson et al., 2024; Rizzone et al., 1994; Townsend et al., 2023), contributing to social withdrawal and emotional overload. The emotional suppression required to maintain daily functioning heightens vulnerability and exhaustion. Similar patterns are reported in other complex conditions, where prolonged uncertainty and threat to the child’s survival amplify pervasive fear and relational disorganization (Assalone et al., 2024; Nonose et al., 2024; Poh et al., 2025). Macro-level determinants like scar-related stigma, language barriers, socioeconomic hardship, and moral interpretations of the accident, intensify stress and hinder treatment adherence (Kairuz et al., 2023; Kornhaber et al., 2018; Rangraz Jeddi et al., 2023). Within a bioecological perspective (Bronfenbrenner and Morris, 2006), these factors constitute contextual pressures that interfere with family reorganization and emotional stability.

Parents mobilize highly dynamic coping strategies, contingent on their threat perception and sense of control (Lazarus and Folkman, 1984). During hospitalization, emotion-focused coping predominates (Egberts et al., 2018; Foster et al., 2017; Schei et al., 2025). Empathic communication by the multidisciplinary pediatric burn team acts as a critical emotional regulator, reducing uncertainty and fostering a sense of safety (Haag and Landolt, 2017; Lernevall et al., 2023; Pan et al., 2018). These findings are consistent with the principles of Trauma-Informed Care, which recognize that burn injuries and repeated medical procedures may be experienced as traumatic events for both children and their carers (Forkey et al., 2021; Simons et al., 2022). Within this approach, trauma-informed communication, early recognition of parental distress, and timely psychosocial support when needed are important components of family-centered pediatric burn care (Forkey et al., 2021; Simons et al., 2022). Evidence from related clinical contexts emphasizes that clear, consistent communication reduces threat perception and promotes adaptive coping (Jenkins et al., 2024). After discharge, trajectories diverge: some parents experience persistent fear, hypervigilance, and ruminative thoughts (Egberts et al., 2020; Wickens et al., 2024), while others report gradual acceptance and emotional reorganization supported by self-care and mindfulness (Huo et al., 2024; Morley et al., 2017; Öster et al., 2014). These patterns mirror prolonged stress responses in parents of children with complex conditions (Yagiela et al., 2019) and align with recent evidence linking threat and control appraisals to the type of coping activated (Peng et al., 2025).

Problem-focused coping intensifies as clinical predictability increases and parents acquire care-related competencies. Active involvement in technical procedures, supervised skill training, competence validation, information-seeking, and coordinated support from the multidisciplinary pediatric burn team are crucial for restoring parents’ sense of control and preparing them for discharge (McGarry et al., 2013; Phillips and Rumsey, 2008; Thompson et al., 2022; Van Niekerk et al., 2020). This trajectory aligns with the principles of Family-Centered Care and care partnerships, wherein shared information and joint decision-making strengthen parental competence and reduce uncertainty across the hospital–home continuum (Kuo et al., 2012; Seniwati et al., 2023). Similar dynamics are reported in pediatric oncology and other care-intensive settings, underscoring the value of structured training and post-discharge follow-up (Shyman et al., 2026). Peer networks and empowerment programs further strengthen confidence and self-efficacy (Mohammadzadeh et al., 2023; Schei et al., 2025). Meaning-making processes that involve spirituality, faith, rituals, and family narratives, emerge as mechanisms for reconstructing coherence and hope (Lernevall et al., 2020; Rose et al., 2005; Suurmond et al., 2020). Depending on the context, these can serve as protective resources or exacerbate distress when intertwined with guilt or punitive interpretations (Khan et al., 2024; Willebrand and Sveen, 2016b). This process is particularly salient after discharge, when families seek to integrate the experience into a “new normal” (Poh et al., 2025).

Non-adaptive responses, such as avoidance, social withdrawal, hypervigilance, and self-blame, appear during both hospitalization and post-discharge phases (Blakeney et al., 1993; Egberts et al., 2019; Wickens et al., 2024), particularly when the perceived threat exceeds available resources.

The transition from hospital to home constitutes a critical juncture, when relief at discharge coexists with vulnerability, fear, and heightened parental responsibility (Santos et al., 2024; Wickens et al., 2024). Other clinical fields describe this stage as emotionally ambivalent and operationally disordered, requiring substantial reorganization of family routines and continuous negotiation between caregiving, work, and daily life (Olson et al., 2024; Van de Riet et al., 2023).

The findings highlight persistent gaps in discharge preparation, including generic verbal instruction, limited supervised practice, and lack of competence validation (Cioga et al., 2024; Lernevall et al., 2021; Öster et al., 2014). These shortcomings are linked to feelings of insecurity, unplanned healthcare contacts, and increased risk of avoidance or errors at home. Evidence from other care contexts shows a similar pattern: absence of structured training heightens fear of mistakes, defensive behaviors, and enduring parental stress (Dewan et al., 2025; Shyman et al., 2026). Structured interventions (including multimodal education, early tele-follow-up, and hybrid care models) can mitigate uncertainty and strengthen confidence (Frederick et al., 2025; Lesher et al., 2023; Mondor et al., 2024). The teach-back method emerges as an essential strategy for consolidating skills and enhancing safety (Dantic, 2014; Talevski et al., 2020). The content and delivery of discharge education, discharge readiness and engagement with follow-up are also shaped by families’ individual contexts. Language barriers may hinder parents’ understanding of discharge recommendations and communication with healthcare professionals, whereas socioeconomic constraints can limit access to rehabilitation services, follow-up appointments, and the resources required to support home care. Stigma associated with burn injuries and visible scarring may discourage social participation and engagement with ongoing support, while cultural beliefs influence how families interpret the injury, seek support, and participate in care (Bakker et al., 2013a; Bayuo and Wong, 2021; Carvalho et al., 2008; Cioga et al., 2024; Coy et al., 2019; Khan et al., 2024; Kornhaber et al., 2018; Lernevall et al., 2023; Öster et al., 2014; Patterson et al., 1993; Rangraz Jeddi et al., 2023; Rose et al., 2005; Rossi et al., 2005; Suurmond et al., 2020; Thompson et al., 2022; Wickens et al., 2024; Woolard et al., 2021). Recent evidence also highlights that equitable discharge planning should be family-centered, adapted to families’ linguistic and social contexts, and supported by communication strategies that promote understanding and continuity of care (Forkey et al., 2021; Glick et al., 2024). Successful implementation of these interventions requires coordinated input from the multidisciplinary pediatric burn team, including professionals involved in clinical care, psychosocial support, rehabilitation, and discharge planning.

Following discharge, family reintegration depends on effective coordination among the multidisciplinary hospital team, primary care, schools, and community services. Peer networks, empowerment initiatives, and structured school reintegration plans promote normalization and reduce isolation (Schei et al., 2025; Van Niekerk et al., 2020). Nonetheless, socioeconomic and cultural barriers heighten vulnerability and risk of exclusion (Kairuz et al., 2023; Townsend et al., 2023), highlighting that parental adjustment extends beyond the family microsystem and is shaped by broader structural conditions (Glick et al., 2024; Harmuch et al., 2026).

Despite substantial evidence, there are notable gaps. Longitudinal studies are limited, restricting understanding of how parental distress, coping and family functioning evolve over time. Fathers, ethnic minority families and siblings remain underrepresented, limiting understanding of the diversity of family experiences. Descriptions of discharge preparation and follow-up pathways are heterogeneous and often insufficient, particularly regarding coordination across care settings. Variation in psychological measures, including frequent use of non-validated tools, further hinder comparability across studies. These gaps highlight the need for robust, longitudinal and equity-oriented research.

Overall, the findings of this review indicate that parental adjustment is a dynamic process that evolves throughout the hospital-to-home continuum. As parents’ emotional responses, coping strategies, and support needs change over time, care should also adapt to these evolving needs. This reinforces the importance of multidisciplinary, family-centered care that extends beyond hospitalization and promotes coordinated support across hospital services, primary care, schools, and community services throughout the child’s recovery trajectory. Within this multidisciplinary approach, pediatric psychologists work collaboratively with nurses and other members of the pediatric burn care team to address the evolving needs of children and families. Their contribution may include caregiver screening, trauma-informed education and support, consultation with the burn care team, and referral for evidence-based psychological intervention when distress persists or interferes with caregiving and family functioning.

### 4.1 Strengths and limitations

This scoping review adhered to the Joanna Briggs Institute (JBI) and PRISMA-ScR guidelines, ensuring methodological rigor and transparency. The broad, multilingual search conducted across eight databases over three decades enhanced representativeness and minimized evidence loss. Inclusion of qualitative, quantitative, and mixed-methods studies enabled integration of experiential depth with psychological and functional indicators, providing a comprehensive portrayal of parental adjustment. The findings offer practical guidance for family-centered care, including competency-based education (teach-back), stratified continuity of care, multimodal educational strategies, psychosocial screening, and equity-oriented approaches, such as cultural mediation and school support.

However, methodological heterogeneity limited comparability and precluded a meta-analysis. Furthermore, no critical appraisal of the included studies was undertaken, in line with JBI guidance for scoping reviews. Consequently, the findings should be interpreted as a mapping of the available evidence rather than an assessment of its methodological quality or strength. Although review articles were included, they were synthesized narratively alongside primary studies without quantitative aggregation, thereby minimizing the potential for double-counting evidence. In addition, gray literature was not systematically searched or included, which may have resulted in the omission of relevant unpublished or non-indexed evidence. The predominance of studies from high-income countries constrains applicability to resource-limited contexts. The focus on parental perspectives excluded siblings, grandparents, and the children themselves. The exclusion of non-accidental burns and studies published in additional languages may have omitted vulnerable subgroups.

### 4.2 Implications for future research

Future priorities include multicenter longitudinal studies to map adjustment trajectories and identify predictors of recovery, persistent distress, and post-traumatic growth. As this scoping review was designed to map the available evidence, it does not allow conclusions regarding the effectiveness of the interventions identified. Pragmatic trials are needed to evaluate interventions, such as competency-based discharge preparation, hybrid follow-up models, and integrated psychosocial screening. Research should incorporate broader family perspectives, consistent with bioecological and family-centered frameworks. Greater representation of low- and middle-income settings is essential, with attention to structural determinants, health literacy, and stigma. Economic evaluations should assess cost-effectiveness of interventions. Finally, the development of a standardized core outcome set for caregiver- and family-centered outcomes in pediatric burns is urgently needed.

## 5 Conclusion

This scoping review highlights that parental adjustment following a child’s burn injury is a continuous and multidimensional process that evolves across the hospital–home continuum. By integrating evidence across this trajectory, the review shows how emotional experiences, family reorganization, parenting skills, and continuity of care interact to shape family adjustment over time. Clear communication, skills-based preparation, and structured follow-up emerged as central elements for promoting safety, trust, and family reorganization. Culturally safe interventions and structured community support may help reduce inequalities and promote positive outcomes across the continuum of care.

## Data Availability

The original contributions presented in the study are included in the article/Supplementary material, further inquiries can be directed to the corresponding authors.

## Footnotes

1
https://doi.org/10.17605/OSF.IO/754ZP
