# Supplementary material for: Parenting experiences and family dynamics in pediatric burn care settings from hospitalization to the return home: a scoping review
Source: Front Psychol. 2026 Sep 23;17:1897592. doi: 10.3389/fpsyg.2026.1897592 (PMC13642316; doi:10.3389/fpsyg.2026.1897592)
Supplement: Supplementary file 1 [file Table_1.docx]

**Supplementary File S1 -** Detailed search strategy according to the databases

| Database | Research strategy |
| --- | --- |
| Medline | ("parents"[MeSH Terms] OR ("famil*"[Title/Abstract] OR "relative*"[Title/Abstract] OR "career*"[Title/Abstract] OR "caregiv*"[Title/Abstract] OR "family care"[Title/Abstract] OR "parent*"[Title/Abstract]) AND ("child*"[Title/Abstract] OR "paediatric"[Title/Abstract] OR "young"[Title/Abstract] OR "youth"[Title/Abstract] OR "adolesc*"[Title/Abstract] OR ("child"[MeSH Terms] OR "child, hospitalized"[MeSH Terms]) AND ("burn*"[Title/Abstract] OR "scald*"[Title/Abstract] OR "thermal injur*"[Title/Abstract] OR "burn wound*"[Title/Abstract] OR "Burns"[MeSH Terms]) AND ("parent role"[Title/Abstract] OR "challenge*"[Title/Abstract] OR "need*"[Title/Abstract] OR "demand*"[Title/Abstract] OR "support*"[Title/Abstract] OR "experience*"[Title/Abstract] OR "perception*"[Title/Abstract] OR "impact"[Title/Abstract] OR "coping"[Title/Abstract] OR "adjust*"[Title/Abstract] OR ("coping skills"[MeSH Terms] OR "adaptation, psychological"[MeSH Terms] OR "psychological adaptation"[Title/Abstract] OR "life change events"[MeSH Terms] OR "life change events"[Title/Abstract]) AND ("Hospitals"[MeSH Terms] OR "home care services, hospital based"[MeSH Terms]) AND ("burn unit"[Title/Abstract]) OR "paediatric hospital"[Title/Abstract] OR "burn center*"[Title/Abstract] OR "burn care center*"[Title/Abstract] OR "paediatric intensive care unit"[Title/Abstract] OR "intensive care units, paediatric"[Mesh Terms] OR "burn care service"[Title/Abstract]) |
| CINAHL | ([MH "Parents" OR TX parent* OR TX famil* OR TX relative* OR TX caregiver* OR TX carer* OR TX "family care"](https://research.ebsco.com/search/results?db=ccm&expanders=concept&facetFilter=databases%3AY2Nt%2CLanguage%3AZW5nbGlzaA%3D%3D%26cG9ydHVndWVzZQ%3D%3D%26c3BhbmlzaA%3D%3D&limiters=FT%3AY%2CLA1%3AY%2CRV%3AY&searchMode=boolean&sort=relevance&sqId=sq%3Aeaa9ba96-389e-4f86-a1ec-df0d4e49b3a8&userDirectAction=true) AND [MH "Child" OR MH "Child, Hospitalized" OR TX child* OR TX pediatric* OR TX paediatric* OR TX young OR TX youth OR TX adolesc*](https://research.ebsco.com/search/results?db=ccm&expanders=concept&facetFilter=databases%3AY2Nt%2CLanguage%3AZW5nbGlzaA%3D%3D%26cG9ydHVndWVzZQ%3D%3D%26c3BhbmlzaA%3D%3D&limiters=FT%3AY%2CLA1%3AY%2CRV%3AY&searchMode=boolean&sort=relevance&sqId=sq%3A7fef7dfa-9302-480b-9c09-d0ef4dbe4972&userDirectAction=true)) AND ([MH "Burns" OR TX burn* OR TX scald* OR TX "thermal injur*" OR TX "burn wound*"](https://research.ebsco.com/search/results?db=ccm&expanders=concept&facetFilter=databases%3AY2Nt%2CLanguage%3AZW5nbGlzaA%3D%3D%26cG9ydHVndWVzZQ%3D%3D%26c3BhbmlzaA%3D%3D&limiters=FT%3AY%2CLA1%3AY%2CRV%3AY&searchMode=boolean&sort=relevance&sqId=sq%3A87dd8422-c2bc-4e36-940e-ecbd4dd6a7b4&userDirectAction=true) AND [TX "parent role" OR TX "parental experience*" OR TX "parental stress" OR TX "parental burden" OR TX coping OR TX adjustment OR MH "Coping Skills" OR MH "Adaptation, Psychological"](https://research.ebsco.com/search/results?db=ccm&expanders=concept&facetFilter=databases%3AY2Nt%2CLanguage%3AZW5nbGlzaA%3D%3D%26cG9ydHVndWVzZQ%3D%3D%26c3BhbmlzaA%3D%3D&limiters=FT%3AY%2CLA1%3AY%2CRV%3AY&searchMode=boolean&sort=relevance&sqId=sq%3A7879de31-051f-4171-b51c-b274b9ed2fae&userDirectAction=true)) AND (MH "Home Care Services, Hospital Based" OR TX "burn unit" OR TX "burn center*" OR TX "burn care center*" OR TX "paediatric intensive care unit" OR TX "burn care service") |
| Scopus | TITLE-ABS-KEY("hospitalized child" OR "pediatric hospital" OR "burn center" OR "burn care center" OR "pediatric intensive care unit" OR "burn care service" OR "hospital discharge" OR "home care") AND TITLE-ABS-KEY("parent role" OR challenge* OR need* OR demand* OR support* OR experience* OR perception* OR coping OR adjustment) AND TITLE-ABS-KEY(parent* OR family OR relatives OR caregiver* OR "family care" OR parenting) AND TITLE-ABS-KEY(child* OR children OR pediatric* OR paediatric* OR young OR youth OR adolesc*) AND TITLE-ABS-KEY(burn OR burns OR scald* OR "thermal injury" OR "thermal injuries" OR "burn wound") |
| Academic Search Complete | (MH "Child, Hospitalized" OR "pediatric hospital" OR "burn center" OR "burn care center" OR "pediatric intensive care unit" OR "hospital discharge" OR "home care") AND (MH "Parents" OR "parental experience" OR "lived experience" OR "parental stress" OR "parental burden" OR "coping mechanisms" OR "parental adaptation" OR "psychological impact" OR "emotional response" OR "perceived support") AND (MH "Child" OR MH "Adolescence" OR children OR child* OR pediatric* OR paediatric* OR young OR youth OR adolesc*) AND (MH "Burns" OR burn OR scald OR "thermal injury" OR  "burn wound") |
| Psychology and Behavioral Science Collection | (DE "Parents" OR "parental experience" OR "lived experience" OR "parental stress" OR "parental burden" OR "coping mechanisms" OR "parental adaptation" OR "psychological impact" OR "emotional response" OR "perceived support") AND (DE "Hospitalized Children"  OR "pediatric hospital" OR "burn center" OR "burn care center" OR "pediatric intensive care unit" OR "hospital discharge" OR "home care") AND (DE "Children" OR DE "Adolescence" OR children OR child* OR pediatric* OR paediatric* OR young OR youth OR adolesc*) AND (DE "Burns" OR burn OR scald OR "thermal injury"OR "burn wound") |
| Web of Science (Clarivate) | ((TS=("Child, Hospitalized" OR "Hospitals" OR "Home Care Services, Hospital-Based")) AND TS=("pediatrics burn unit" OR "pediatric hospital" OR "burn center" OR "burn care center" OR "pediatric intensive care unit" OR "burn care service" OR "hospital discharge" OR "home care")) AND ((TS=("parent role" OR challenge OR need OR demand OR support OR experiences OR perception* OR coping OR adjustment)) AND TS=("Parents" OR family OR relatives OR career OR caregiver OR "family care in" OR parents OR parenting)) AND ((TS=(children OR child* OR paediatric OR pediatric OR young OR youth OR adolesc*)) AND TS=(burn OR scald OR "thermal injury" OR "thermal injuries" OR "burn wound"))) |
| Cochrane Library | (parent* OR family OR caregiver*) AND (child* OR pediatric* OR paediatric* OR adolescent*) AND (burn OR burns OR scald* OR "thermal injury") AND (hospital* OR "burn center" OR "intensive care" OR "hospital discharge" OR "home care") |

\# = Research line; * = Truncation
