# Supplementary material for: Parenting experiences and family dynamics in pediatric burn care settings from hospitalization to the return home: a scoping review
Source: Front Psychol. 2026 Sep 23;17:1897592. doi: 10.3389/fpsyg.2026.1897592 (PMC13642316; doi:10.3389/fpsyg.2026.1897592)
Supplement: Supplementary file 2 [file Table_2.docx]

**Supplementary File S2.** Operationalisation of Braun and Clarke's thematic analysis and generation of analytical themes

| **Braun & Clarke phases** | **How the phase was operationalised in this scoping review** | **Examples from the analytical process** | **Output** |
| --- | --- | --- | --- |
| **1. Familiarisation with the data** | The extracted findings from all included studies were read repeatedly and compared to achieve an in-depth understanding of parents' and families' experiences across hospitalisation and the post-discharge period. Data were examined across emotional, family, contextual, coping and healthcare-related dimensions. | Examples of recurring findings include shock, fear, guilt, anxiety, changes in family roles, caregiver burden, communication with healthcare professionals, discharge experiences, continuity of care and reintegration into everyday life. | Comprehensive understanding of the dataset and identification of recurrent patterns across studies. |
| **2. Generating initial codes** | Relevant findings were coded using both deductive and inductive approaches. Deductive coding was informed by the review objective, whereas with inductive coding new concepts emerged from the studies. Similar findings were progressively grouped according to conceptual similarity. | Examples of initial codes include fear and uncertainty, parental guilt, psychological distress, family role changes, communication with healthcare professionals, participation in care, preparation for discharge, social support, stigma, school reintegration, spiritual coping, avoidance and hypervigilance. | Initial coding framework representing the diversity of parents' and families' experiences. |
| **3. Searching for themes** | Related codes were organised into broader thematic categories through iterative comparison across studies. At this stage, the emphasis was on identifying recurring patterns rather than producing the final thematic structure. | Seven preliminary thematic categories were identified: (1) Emotional Impact and Parental Experience; (2) Coping Strategies Used; (3) Relationship with the Healthcare Team; (4) Transition to a New Caregiving Role; (5) Family Relationships; (6) Contextual Factors; and (7) Acceptance. | Seven preliminary thematic categories. |
| **4. Reviewing themes** | The preliminary thematic categories were repeatedly reviewed and compared across the complete dataset to assess internal coherence and external distinction. Categories showing conceptual overlap were merged and reorganised following discussions within the research team. | Family Relationships and Contextual Factors were integrated with the broader traumatic impact of paediatric burns. Relationship with the Healthcare Team and Transition to a New Caregiving Role were reorganised around discharge preparation and continuity of care. The preliminary categories were consolidated into four broader analytical themes. | Four refined analytical themes. |
| **5. Defining and naming themes** | The four analytical themes were further refined to ensure conceptual clarity and alignment with the review objective. During this phase, *Family Adjustment and Acceptance* was reconsidered. Rather than representing a distinct analytical domain, adjustment consistently emerged across emotional responses, coping processes and discharge experiences. Consequently, it was conceptualised as a transversal process underpinning the thematic structure. | Final analytical themes: (1) Traumatic Impact of Paediatric Burns and Consequences for Family Dynamics; (2) Coping Strategies of Parents and Families; and (3) Preparation for Discharge and Continuity of Care. Family adjustment and acceptance were interpreted as transversal processes reflected across all three themes. | Three final analytical themes and corresponding subthemes. |
| **6. Producing the report** | The final thematic structure was reported in the Results section, supported by evidence from the included studies and presented according to the review objective. The Discussion subsequently interpreted these findings in relation to the wider literature and theoretical frameworks. | Themes illustrated with evidence from hospitalisation and post-discharge studies. | Results and Discussion presented in the manuscript. |
