# Supplementary material for: Parenting experiences and family dynamics in pediatric burn care settings from hospitalization to the return home: a scoping review
Source: Front Psychol. 2026 Sep 23;17:1897592. doi: 10.3389/fpsyg.2026.1897592 (PMC13642316; doi:10.3389/fpsyg.2026.1897592)
Supplement: Supplementary file 3 [file Table_3.docx]

**Supplementary File S3.** Summary of Included Studies (n = 63)

| **Author/Year** | **Country** | **Study Design** | **Aim(s)** | **Setting** | **Main Findings** |
| --- | --- | --- | --- | --- | --- |
| **Wickens et al. [3]** | Australia | Qualitative study | To explore psychological trauma associated with paediatric burns from the mothers’ perspectives. | Hospital burn unit + home (recovery) | Mothers described intense trauma, guilt and hypervigilance. The transition home amplified emotional challenges; supportive professional relationships facilitated recovery. |
| **Atapattu et al. [4]** | Australia | Quantitative study | To examine predictors of long-term PTSS in parents and the relationship between parent PTSS and child QoL after burns. | Paediatric burn unit + post-discharge follow-up | Parental PTSS remained high over time. Early anxiety and injury features predicted later distress; parental PTSS was associated with poorer child QoL. |
| **Egberts et al. [5]** | Netherlands | Quantitative study | To examine longitudinal associations between mothers’ emotional responses following paediatric burn injury and subsequent posttraumatic stress symptoms and depressive symptoms up to 18 months postburn | Hospital | Maternal posttraumatic stress symptoms and depressive symptoms were strongly associated with early negative emotional responses, particularly guilt, fear and threat appraisals. Symptoms were highest shortly after the burn event and decreased over time; however, a substantial subgroup of mothers showed persistent clinically relevant posttraumatic stress and depressive symptoms up to 18 months postburn |
| **Santos et al. [6]** | Portugal | Qualitative study | To explore parents’ experiences during their child’s hospitalization for a burn injury and inform an integrated care model. | Paediatric burn centre | Parents reported distress, fear, uncertainty, and a need for continuous communication and emotional support. Nurses played a key role in helping parents feel safe and informed. |
| **Lernevall et al. [7]** | Norway | Qualitative study | To gain an in-depth understanding of parents’ lived experience caring for their burn-injured child at home. | Home setting | Parents faced persistent fear of complications, anxiety during wound care, and emotional exhaustion. Home care required practical competence and emotional resilience; ongoing guidance from nurses was crucial. |
| **Huo et al. [8]** | China | Quantitative study | To examine the association between perceived stress and anxiety, depression, and sleep quality in parents of children with burns, and test the moderating effect of social support. | Paediatric burn unit (Wuhan) | High parental stress was associated with worse mental health. Social support significantly moderated negative outcomes, highlighting the need for systematic psychological screening. |
| **Khan et al. [9]** | Pakistan | Qualitative study | To explore the multifaceted challenges faced by parents of paediatric burn survivors. | Tertiary burn centre (hospital; post-admission + follow-up) | Parents experienced psychological distress (anxiety, guilt), physical exhaustion, stigma, financial strain, and fear of home care. Coping was emotion-focused and religious. Highlights need for holistic psychosocial and socioeconomic support. |
| **Bayuo & Wong [10]** | Hong Kong | Scoping review | To map existing evidence on challenges experienced by family members of burn patients. | Multiple burn care settings | Identified emotional burden, financial challenges, complex care responsibilities, and insufficient communication with health professionals. Emphasised the need for structured psychosocial and informational support for families. |
| **Kairuz et al. [11]** | Australia | Protocol study | To co-design culturally appropriate informational resources for Aboriginal and Torres Strait Islander families of children with burns. | Paediatric burn unit (Clubbe Ward, NSW) | Protocol anticipating culturally safe co-design methods. Emphasises community partnership, cultural identity, and health literacy as drivers of effective burn-care resources. |
| **Lernevall et al. [12]** | Norway | Qualitative study | To explore parents’ support needs during their child’s hospitalization. | Paediatric burn centre | Parents needed reassurance, emotional containment, clear communication, and participation in care. Emotional support from nurses was central to feeling secure. |
| **Thompson et al. [13]** | United Kingdom | Qualitative study | To describe parents’ experiences administering dressing changes at home following paediatric burn injury. | Home setting (post-discharge), recruited from a children’s hospital | Parents perceived home dressing changes as highly stressful, painful for the child, and emotionally taxing for themselves. Adequate training, clear instructions, and follow-up support were essential for confidence and safe care. |
| **Schei et al. [14]** | Norway | Qualitative study | To explore parental experiences, coping, and the impact of Family Burn Camp after paediatric burn injury. | Post-discharge; community rehabilitation (Family Burn Camp) | Parents described transition from “acute chaos” to a “scarred future”, isolation, and the dual burden of caring while needing help. Camp participation fostered empowerment, peer support, and normalisation of scars. |
| **Coombes et al. [15]** | Australia | Qualitative study | To understand the aftercare journey of First Nations families whose child sustained a burn injury. | Hospital + community aftercare | Families experienced fragmented care pathways, cultural barriers, and logistical burdens. Culturally safe, continuous, community-linked care was identified as essential. |
| **Townsend et al. [17]** | United States | Quantitative study | To identify risk factors for parental PTSS after minor paediatric burns and examine associations with child’s quality of life. | Outpatient paediatric burn clinic (academic medical centre) | Despite minor burns, parental PTSS was common and negatively correlated with child’s quality of life. Key predictors were parent anxiety levels, child pain, and injury-related fears. Recommends routine psychological screening in outpatient care. |
| **Chen et al. [18]** | China | Quantitative study | To evaluate psychological symptoms among parents of hospitalized children with burns and identify associated factors. | Two burn units in Shanghai – during hospitalization | Parents showed high levels of anxiety, depression, and somatization. Burn severity, length of stay, and perceived stress predicted worse outcomes. Social support emerged as a protective factor. |
| **Egberts et al. [19]** | Netherlands | Qualitative study | To explore parents’ intrusive memories and appraisals after their child’s burn injury. | Three Dutch burn centres | Parents reported persistent intrusive memories, guilt, and fear. Appraisals influenced emotional recovery, suggesting early cognitive-focused interventions may prevent chronic distress. |
| **Mohammadzadeh et al. [20]** | Iran | Quantitative study | To evaluate effects of an empowerment model on children's quality of life and parents’ perceived stress. | Three hospitals (Birjand) | Intervention significantly improved children’s QoL and reduced parental stress scores. Reinforces the value of structured, family-centred educational programs in paediatric burns. |
| **Cioga et al. [21]** | Portugal | Discussion paper | To explore the impact of paediatric burns on parents and families and propose ways to support parental adjustment after burn injury. | Hospital + home (acute phase to return home). | Synthesises evidence showing sustained parental emotional burden and highlights the need for integrated psychosocial support across hospitalization and post-discharge care. |
| **Foster et al. [26]** | Australia | Qualitative study | To explore parents’ experiences and needs during the acute hospitalization of their critically injured child (including burns). | Paediatric trauma centres (acute hospital phase) | Parents reported intense distress, uncertainty, and unmet informational needs. Communication quality and emotional support from staff were crucial for parental coping. |
| **Shoghi et al. [27]** | Iran | Qualitative study | To interpret and explain mothers’ lived experiences caring for children with burn-induced disabilities. | Home setting (post-discharge) | Mothers described deep emotional suffering, social stigma, chronic caregiving strain, and loss of personal identity. Supportive family relationships and empathetic professionals were central to coping. |
| **Haag & Landolt [28]** | Switzerland | Quantitative study | To assess acute stress in young children and their parents shortly after a burn injury and explore mediating mechanisms. | Hospital (University Children’s Hospital Zurich) | High acute stress was prevalent in both parents and children. Parental stress partially mediated the effect of injury severity on child stress, underscoring the importance of parental mental health. |
| **Shaygan et al. [29]** | Iran | Quantitative study | To evaluate the effectiveness of a resilience training program in reducing anxiety in mothers and hospitalized children with burns. | Paediatric burn unit | The resilience program significantly reduced maternal and child anxiety, supporting early psychological intervention during hospitalization. |
| **Van Niekerk et al. [30]** | South Africa | Qualitative study | To explore psychosocial recovery enablers for children with burns, from the perspective of children, caregivers, and burn staff. | Paediatric burns unit + home + school environments | Recovery was influenced by family cohesion, school reintegration experiences, community perceptions, and access to psychosocial services. Emphasised multisystemic support. |
| **De Young et al. [43]** | Australia | Quantitative study | To examine the course of parent and child acute stress and identify predictors of PTSD. | Hospital + outpatient follow-up | High acute stress was common in both parents and children. Parental acute stress mediated the relationship between burn severity and child PTSD, reinforcing the need to support parents early. |
| **Ravindran et al. [46]** | India | Qualitative study | To explore parents’ experiences of caring for a burn-injured child and the social processes involved. | Hospital (tertiary paediatric burn centre) | Parents experienced strong feelings of guilt, blame, and social stigma, affecting coping and help-seeking. The study highlights the need for culturally sensitive psychosocial support. |
| **Carvalho & Rossi [47]** | Brazil | Narrative literature review | To describe, through a literature review, the impact of burn injury and hospitalization in a burn unit on family dynamics. | Hospital – burn unit. | The review identified that paediatric burns and hospitalization profoundly disrupt family functioning, generating emotional overload, changes in roles, and financial strain. It recommends family-centred nursing care, better preparation for discharge, and structured support to facilitate family adaptation. |
| **McGarry et al. [48]** | Australia | Quantitative study | To assess psychological distress and resilience in parents of paediatric burn survivors. | Tertiary children’s hospital | Parents reported considerable trauma symptoms, influenced by injury severity and subjective threat. Resilience moderated distress, suggesting opportunities for strengths-based interventions. |
| **Pan et al. [49]** | Brazil | Integrative literature review | To synthesise evidence on school reintegration programs for paediatric burn survivors. | School reintegration (post-hospital) | Identified significant gaps in structured school reintegration processes. Children faced stigma, emotional difficulties, and limited institutional support. Highlights need for multidisciplinary collaboration between hospitals, families, and schools. |
| **Mazzi et al. [50]** | Brazil | Qualitative study | To understand the emotional experiences of mothers of burned children after hospital discharge, in the home-care phase. | Outpatient burn clinic, post-discharge (home-care context). | Mothers reported intense suffering, fear of complications and re-injury, insecurity in providing care, and social isolation. The findings stress the importance of continued follow-up, emotional support, and guidance to strengthen mothers’ confidence in home care. |
| **El Hamaoui et al. [51]** | Morocco | Quantitative study | To determine the prevalence of depression and related predictors among mothers of burned children. | Hospital | High maternal depression rates were identified, associated with injury severity and caregiving challenges. |
| **Johnson et al. [53]** | Australia | Qualitative study | To explore how patients and families redefine normality after burn injury during hospitalisation and early recovery. | Burn unit (hospital) + early home recovery | Families experienced a “trauma bubble,” with disrupted routines and fragile adjustment post-discharge. Supportive professionals facilitated the transition toward a new sense of normality. |
| **Öster et al. [54]** | Sweden | Qualitative study | To explore parents’ experiences of how a child’s burn injury affected family life and adjustment. | Post-hospitalization / rehabilitation | Families described long-lasting changes in routines, persistent fear, and challenges with reintegration. Support needs extended well beyond hospital discharge. |
| **Willebrand & Sveen [55]** | Sweden | Quantitative study | To investigate perceived lack of support among parents after their child’s burn injury and associated factors. | Post-hospitalization (two national burn centres) | Many parents felt insufficiently supported, especially regarding psychological needs. Lack of support was associated with greater distress and fear-avoidance beliefs. |
| **Rose et al. [56]** | USA | Quantitative study | To evaluate cultural differences in parental stress among English- and Spanish-speaking parents. | Hospital + Long-term follow-up | Cultural and linguistic factors influenced stress profiles; Spanish-speaking parents reported higher sustained stress. |
| **Kornhaber et al. [57]** | Multiple countries | systematic review -Qualitative | To synthesise experiences of guilt, blame, and shame in people affected by burns, including parents. | Across care settings (hospital, rehabilitation, long-term adjustment) | Burn injuries evoke profound feelings of guilt and shame in parents and caregivers. These emotions strongly influence adjustment, coping, and support-seeking behaviours. Recommends trauma-informed and non-blaming approaches in burn care. |
| **LeDoux et al. [58]** | USA | Quantitative study | To investigate the relationship between parental emotional states, family environment, and child behavioural adjustment. | Hospital | Child adjustment was significantly influenced by family climate, parental emotional wellbeing, and support. |
| **Patterson et al. [60]** | UK | Mixed-methods study | To examine psychological outcomes in young burned children and family functioning over time. | Hospital + Home | Parental distress impacted child adjustment; improvements occurred gradually during follow-up. |
| **Rizzone et al. [61]** | USA | Quantitative study | To identify factors associated with PTSD in mothers of burned children. | Hospital | High PTSD prevalence was found, particularly with severe injuries and greater perceived threat. |
| **Suurmond et al. [62]** | Netherlands | Quantitative study | To investigate guilt, depressive symptoms, and PTSS in Dutch vs ethnic minority parents. | Hospital burn centres | Ethnic minority parents reported significantly higher distress, guilt, and depressive symptoms. Cultural factors influenced help-seeking behaviours. |
| **Bakker et al. [63]** | Netherlands (international scope) | Narrative review | To synthesise research on psychological consequences of paediatric burns for children and their families. | Multiple contexts | The review identifies high rates of PTSD, depression, behavioural problems, and family strain after paediatric burns, and highlights the importance of multidisciplinary psychosocial support. |
| **Morley et al. [64]** | United Kingdom | Qualitative study | To explore mothers’ experiences of their young child’s dressing changes in a burn unit. | Specialist children’s burn unit | Dressing changes were described as traumatic, emotionally distressing, and physically demanding. Mothers required more emotional support and involvement in care planning. |
| **Willebrand & Sveen [65]** | Sweden | Quantitative study | To examine injury-related fear-avoidance beliefs in parents and their association with PTSD symptoms and perceived child health. | Home (nationwide postal survey) | Fear-avoidance beliefs were common and strongly associated with parental PTSD symptoms, independent of burn severity. Highlights the importance of addressing maladaptive beliefs early. |
| **Phillips et al. [66]** | USA | Qualitative study | To identify support needs and concerns of family members of hospitalized burn patients. | Hospital | Key needs included clear communication, emotional support, involvement in care, and adequate information provision. |
| **Smith et al. [67]** | New Zealand | Quantitative study | To compare nurses’ and parents’/caregivers’ perceptions of support interventions offered before, during, and after paediatric burn dressing changes. | Hospital – paediatric burn unit; dressing changes in a specialised burn bathroom. | Important discrepancies emerged between nurses and parents regarding the adequacy of information, preparation, and comfort measures. The study underlines the need to better involve parents in planning procedures, improve communication, and individualise non-pharmacological support during dressing changes. |
| **Rimmer et al. [68]** | USA | Quantitative study | To identify stressful, difficult, and disruptive aspects of paediatric burn hospitalization and follow-up from the parents’ perspective. | Paediatric burn centre (inpatient + outpatient) | Parents highlighted dressing changes, wound care, sleep disruption, and emotional burden as significant stressors. Burn centre staff were found to play a key role in reducing caregiver burden. |
| **Santos et al. [69]** | Portugal | Quantitative study | To analyse parental psychosocial adjustment and child outcomes over time, testing the mediating role of family functioning. | Hospital (baseline) + outpatient follow-up (1, 3, 6 months) | Parental anxiety, depression and PTSS decreased over time; coping improved. Family functioning predicted child outcomes and mediated the effect of parental distress on recovery. |
| **Bakker et al. [70]** | Netherlands & Belgium | Quantitative study | To investigate the trajectory of traumatic stress symptoms in mothers and fathers after their child’s burn injury. | Hospital burn centres (multiple) | Both parents showed high acute stress, with different symptom trajectories. Parental PTSS persisted up to 18 months, emphasizing the need for early identification and support for both mothers and fathers. |
| **Odar et al. [72]** | USA | Quantitative study | To examine prevalence and correlates of PTSS in parents of young children after minor-to-moderate burns. | Outpatient burn clinic | Significant levels of parental PTSS were found even for minor burns. Parental distress was associated with child pain, acute stress, and perceived injury threat. |
| **Egberts et al. [73]** | Netherlands | Qualitative study | To examined occurrence and within-family associations of traumatic stress reactions after child burn injury, while in the same model addressing the role of parents' own symptoms in their reports of child symptoms.. | Three Dutch burn centres | Parents experienced vivid, distressing memories, guilt, and fear of recurrence. Appraisals strongly shaped emotional outcomes. Findings support early interventions targeting maladaptive appraisals. |
| **Rossi et al. [74]** | Brazil | Qualitative study | To explore family members’ cultural perceptions related to hospital discharge after a burn injury. | Hospital | Families experienced stigma, fear, and insecurity regarding home care, underscoring gaps in discharge preparation. |
| **Blakeney et al. [75]** | USA | Quantitative study | To assess parental stress as both a cause and consequence of paediatric burn injury. | Hospital | Parental stress was strongly associated with burn severity and caregiving burden, influencing child recovery. |
| **Heath et al. [76]** | United Kingdom | Mixed methods study | To develop and evaluate a peer-informed website to support parents of children with burns. | Healthcare and support institutions across the UK | Parents valued online, peer-informed resources for emotional support and practical guidance. Accessibility and relevance of information were central to user satisfaction. |
| **Kilburn & Dheansa [77]** | England | Quantitative study | To examine social and economic impacts of children’s burns on families. | Hospital outpatient setting | Families experienced financial burden, work disruption, and emotional strain. Socioeconomic impacts were substantial even in less severe burns. |
| **Phillips & Rumsey [78]** | United Kingdom | Quantitative study | To quantify levels of psychosocial distress in parents of children with burns and to generate evidence-based recommendations for psychosocial support services. | Hospital – specialist burn units (inpatient and outpatient stages). | Parental distress was frequent and clinically relevant. The study showed gaps in access to psychological support and highlighted the need for systematic screening and structured psychosocial interventions for families. |
| **Coy et al. [79]** | United Kingdom | Qualitative study | To map the emotional journeys of families after a child’s burn injury and identify service improvements. | Regional paediatric burns centre (SWCBC) | Emotional trajectories showed prolonged anxiety, guilt, and fear of reinjury. Families emphasised the need for improved communication, psychosocial support, and educational materials. Led to development of co-designed service improvements. |
| **Parrish et al. [80]** | USA | Quantitative study | To examine prevalence and predictors of parental distress following paediatric burn injury. | Regional burn centre – outpatient clinic | A substantial proportion of parents reported clinically significant distress. Predictors included child pain, injury severity, and parent psychological history. Recommends routine screening. |
| **Rangraz Jeddi et al. [81]** | Iran | Quantitative study | To assess healthcare needs, eHealth literacy, mobile phone use, and intention to use digital tools for self-management among caregivers of burn-injured children. | Burn centre (inpatient + outpatient) | Caregivers demonstrated moderate eHealth literacy but high interest in mobile-based tools. Key needs included wound-care guidance, psychological support, and easier access to professionals. Highlights opportunity for digital health interventions. |
| **Lernevall et al. [82]** | Norway | Integrative review | To synthesise evidence on support needs of parents of hospitalized children with burns. | Hospital settings (international) | Parents consistently needed emotional support, clear communication, involvement in care, and guidance for discharge. Highlights universal parental concerns across countries. |
| **Jopke et al. [83]** | USA | Case study | To compare outcomes of two children with severe burns, one of whom received essential oils as complementary therapy. | Paediatric ICU | The child receiving essential oils showed reduced anxiety and improved comfort; however, findings are limited by small sample size and exploratory design. |
| **Tyack & Ziviani [84]** | Australia | Quantitative study | To identify predictors of functional outcomes in children 6 months post-burn. | Hospital + Follow-up | Functional recovery was predicted by burn severity, child premorbid factors, and parental stress levels. |
| **Bakker et al. [85]** | Netherlands & Belgium | Quantitative study | To examine behavioural and emotional problems in preschool children post-burn and their relationship with parental distress. | Hospital burn centres | Children showed elevated behavioural problems at 3 and 12 months. Parental distress strongly predicted child emotional outcomes. |
| **Özer & Vural [86]** | Turkey | Quantitative study | To identify problems experienced by families after discharge and their causes. | Burn clinic – post-discharge | Families reported insufficient knowledge, wound-care difficulties, financial strain, and emotional distress. Findings reinforce the need for structured discharge preparation. |
| **McGarry et al. [87]** | Australia | Qualitative study | To describe parents’ experiences of their child’s hospitalization for a burn injury. | Hospital (tertiary paediatric hospital) | Parents reported fear, emotional overload, and loss of control. They emphasised the importance of clear communication and supportive nurse–parent partnerships to reduce anxiety and promote coping. |
